# Computational Analysis and Biological Activities of Oxyresveratrol Analogues, the Putative Cyclooxygenase-2 Inhibitors

**DOI:** 10.3390/molecules27072346

**Published:** 2022-04-06

**Authors:** Nathjanan Jongkon, Boonwiset Seaho, Ngampuk Tayana, Saisuree Prateeptongkum, Nongnaphat Duangdee, Panichakorn Jaiyong

**Affiliations:** 1 Department of Social and Applied Science, College of Industrial Technology, King Mongkut’s University of Technology North Bangkok, Bangkok 10800, Thailand; nathjanan.j@cit.kmutnb.ac.th; 2 Department of Chemistry, Faculty of Science and Technology, Thammasat University, Pathum Thani 12120, Thailand; boonwiset.seah@dome.tu.ac.th (B.S.); saisuree@tu.ac.th (S.P.); 3 Drug Discovery and Development Center, Office of Advance Science and Technology, Thammasat University, Pathum Thani 12120, Thailand; ngampuk@tu.ac.th

**Keywords:** inflammation, molecular docking, protein–ligand interactions, quantum chemistry, stilbenoid

## Abstract

Polyphenols are a large family of naturally occurring phytochemicals. Herein, oxyresveratrol was isolated from ethanolic crude extracts of *Artocarpus lacucha* Buch.-Ham., and chemically modified to derive its lipophilic analogues. Biological screening assays showed their inhibitory potency against cyclooxygenase-2 (COX-2) with very low cytotoxicity to the MRC-5 normal cell lines. At the catalytic site of COX-2, docking protocols with ChemPLP, GoldScore and AutoDock scoring functions were carried out to reveal hydrogen bonding interactions with key polar contacts and hydrophobic pi-interactions. For more accurate binding energetics, COX-2/ligand complexes at the binding region were computed in vacuo and implicit aqueous solvation using M06-2X density functional with 6-31G+(d,p) basis set. Our computational results confirmed that dihydrooxyresveratrol (**4**) is the putative inhibitor of human COX-2 with the highest inhibitory activity (IC_50_ of 11.50 ± 1.54 µM) among studied non-fluorinated analogues for further lead optimization. Selective substitution of fluorine provides a stronger binding affinity; however, lowering the cytotoxicity of a fluorinated analogue to a normal cell is challenging. The consensus among biological activities, ChemPLP docking score and the binding energies computed at the quantum mechanical level is obviously helpful for identification of oxyresveratrol analogues as a putative anti-inflammatory agent.

## 1. Introduction

Inflammation is the intrinsic immune response triggered by injury and infection. At the sites of tissue damage, the biosynthesis of prostaglandins in such inflamed tissue is significantly increased [1]. Prostaglandins—the lipid prostanoids derived from arachidonic acid—play a key role in innate immunity and inflammatory response. One of the major pathways of prostaglandin regulation in vivo involves the catalytic process of cyclooxygenase (COX) enzymes, of which three isoforms have been identified and are referred to as COX-1, COX-2 and the COX-1 splice variant, COX-3. COX isozymes catalyse the oxygenation of polyunsaturated fatty acids at the cyclooxygenase active site, and reduce the produced hydroperoxide to an alcohol at the peroxidase active site [2]. The basis of their difference is hydroperoxide-dependent activation; COX-2 approximately requires lower hydroperoxide level by a 10-fold amount [3]. COX-1 and COX-2 isoforms share approximately 60% sequence homology of amino acids with similar shape of the catalytic region [4,5]. However, the substitution of isoleucine numbered 523 in COX-1 with valine in COX-2 enlarges the volume of the COX-2 active site by approximately 25% [4,5]. It is worth noting that this valine residue at the pocket of COX-2 in this work is numbered 509 according to the originally retrieved protein structure of 3LN1 as a PDB entry code. Other subtle changes also result in a wider channel opening by 17% in COX-2 [5]. In addition, both COX isoforms differ in their pattern of regulation [6]. COX-1 acts as a housekeeping enzyme to maintain homeostasis such as gastric epithelial cytoprotection, kidney function and platelet aggregation; on the other hand, COX-2 is involved in inflammation [7]. Therefore, COX-2 has become a target of anti-inflammatory therapeutics. Deleterious side effects can occur as a consequence of drug administration used to treat inflammatory conditions if the administered drugs synergistically suppresssed the functions of COX-1.

The design of novel inhibitors to selectively bind to a specific COX isoform with less harmful adverse effects has been a challenge. Most of the FDA-approved nonsteroidal anti-inflammatory drugs (NSAIDs) such as diclofenac, ibuprofen, indomethacin, naproxen, oxaprozin and piroxicam are nonselective, inhibiting both COX-1 and COX-2 isoforms, and are associated with well-known side effects. Recent progress in designing coxibs as a COX-2 selective NSAID has been addressed in six broad categories [8,9]. Only celecoxib remains legally available for use after rofecoxib and valdecoxib were withdrawn due to their cardiotoxicity [10]. Gastrointestinal side effects and cardiovascular thrombotic risk of celecoxib and other COX-2-selective drugs have also been reported [11,12]; thus, the use of celecoxib in patients with cardiovascular problems should be avoided [13]. As an alternative to coxibs, medicinal plants exhibit potent anti-inflammatory properties [14] due to a variety of their phytochemicals including phenolics [14,15,16,17], terpeniods [18,19], alkaloids [20] and flavonoids [17,21,22].

**Figure 1:**
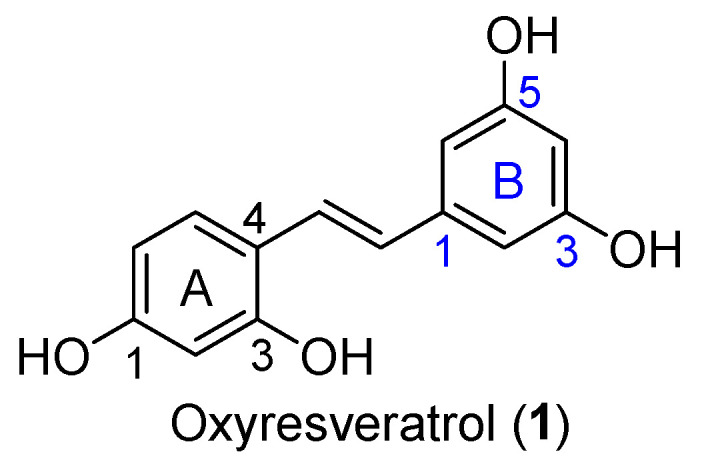
Chemical structure of oxyresveratrol (*trans*-2,3′,4,5′-tetrahydroxystilbene).

Phenolic compounds are a major group of secondary plant metabolites and exhibit strong antioxidative, anticarcinogenic and anticyclooxigenase properties [23]. Stilbene-based phenolic compounds are widely found in nature with a diverse array of pharmacological and biochemical properties [24,25,26]. *Trans*-stilbenoids—the polyphenolic stilbene—are more stable and biologically relevant mediators with acidic and amphiphilic properties [8]. Oxyresveratrol (*trans*-2,3′,4,5′-tetrahydroxystilbene (**1**), Figure 1) is a natural stilbenoid found in the root, stem and heartwood of plants including *Artocarpus heterophyllus* L. (Moraceae) [27], *Scirpus maritimus* (Cyperaceae) [28] and *Morus alba* L. (Moraceae) [29]. It has been brought to our attention due to a broad spectrum of biological activities such as antioxidant [30], anti-tyrosinase [31], antiviral [32], neuroprotective [27] and anti-inflammatory activities [33,34]. Interestingly, the anti-inflammatory activity of oxyresveratrol has been mentioned via various pathways such as the COX enzyme, nitric oxide synthase (iNOS) and NF-*κ*B activation [33]. It has been reported that oxyresveratrol reduced not only paw edema induced by carrageenan in rats [35], but also mRNA expression levels of COX-2 isoform [36]. It can inhibit a 5-lipoxygenase (5-LO) enzyme, which induced inflammatory mediators named leukotrienes (LTs) for asthma control, with the half-maximal inhibitory concentration (IC_50_) in µM [34].

Despite the biological activity of COX inhibitors reported in the literature [37], insufficient metabolic stability and a high degree of non-specific binding have been the issues for consideration in lead optimization. Incorporation of fluorine into stilbene-like compounds is one effective way to increase their lipophilicity, resulting in an increase in metabolic stability and membrane permeation. Electron withdrawal by fluorine introduces a strong polarization of the C-F bond, which pronounces the electronic effects for drug-target interactions as in the case of celecoxib—the fluorine-containing clinical NSAID and a selective COX-2 inhibitor [38]. Thus, we introduced fluorine atom(s) to the double bond of an oxyresveratrol scaffold, yielding the fluorinated oxyresveratrol analogues.

To explore ligand–protein interactions, molecular docking is one of the most used approaches in structure-based drug design, which requires the three-dimensional structure of a macromolecular target [39,40]. The docking process involves: (i) sampling or generating a set of all possible ligand conformations and orientations; and (ii) evaluating the binding affinity of each ligand pose placed at the active site of its target. Therefore, top-ranked poses of docked ligand(s) depend on the quality of the search algorithm and scoring function. Different sampling algorithms such as shape matching, systematic, stochastic and simulation methods can be applied for a conformational search [39,41,42]. Then, the binding affinity of each generated pose is estimated and ranked by the scoring functions, which are traditionally classified into the force field-based, knowledge-based, and empirical scoring functions [39,41,42]. Effective scoring functions have been a challenge in molecular docking and often limit their reliability [43,44]. Therefore, quantum mechanical (QM) methods have been increasingly used in drug design due to a demand for the accurate estimation of binding affinities [45,46,47]. Some QM computed parameters, such as a dipole moment and atomic charges, have been found to be useful for improving the accuracy of a docking protocol [43,48].

In this work, we exploit the structure-based computational approaches to investigate the binding interactions of oxyresveratrol and its analogues at the catalytic site of COX-2. Molecular docking procedures with different scoring functions are used to predict the binding modes and affinities of each COX-2/ligand complex. We perform energy calculations on the optimized COX-2 complexes with the best-docked pose using M06-2X density functional and 6-31G(d) basis set in both vacuo and implicit aqueous solvation. The computed binding energetics of complexes are discussed and compared with the cytotoxicity and inhibitory potency screened by using the human COX-2 assay kit. This computational analysis is aimed at providing a more accurate description of the binding affinity of studied oxyresveratrol analogues. 

## 2. Results and Discussion

### 2.1. Binding Affinity of COX-2/Celecoxib Complex

**Figure 2:**
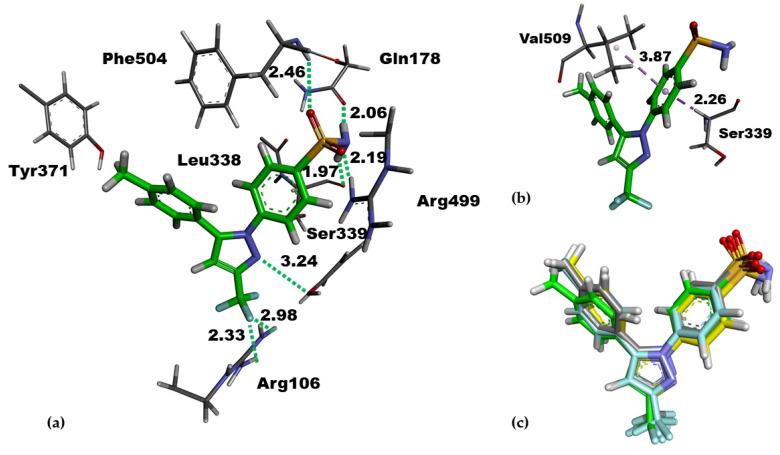
Relaxed pose obtained from a 1 ns molecular dynamics (MD) simulation of celecoxib (stick representation in green). (**a**,**b**) Binding interactions of celecoxib with key amino acids in the catalytic pocket of COX-2. The unit of distance in proximity is angstrom. (**c**) The superposition of the MD structure of celecoxib (green) and the best-docked pose of celecoxib from AutoDock (grey) with the RMSD value of 0.68 Å, ChemPLP (yellow) with the RMSD value of 0.55 Å and GoldScore (Cyan) with the RMSD value of 0.56 Å.

To understand the nature of the catalytic pocket of COX-2, we performed a short molecular dynamics simulation of the complex of COX-2/co-crystallized celecoxib (PDB entry: 3LN1, Figure S1, in the Supplementary Materials) to relax the complex geometry. A long and hydrophobic channel of the cyclooxygenase active site is applicable to NSAIDs for binding [2,49]. Figure 2 illustrated the molecular interactions of a relaxed pose of co-crystallized celecoxib, the known selective COX-2 inhibitor. Its trifluoromethyl group was surrounded by the methyl groups of Val102, Leu345 and Leu517 at the binding entrance formed by Arg106 and Tyr341. These two amino acids appeared to reveal the hydrogen bonds and/or charged interactions with the carboxyl groups of non-selective NSAIDs [11,50,51]. Unlike traditional NSAIDs, celecoxib placed its sulfamoylphenyl moiety in a side pocket located at the polar His75, Glu178 and Arg499 residues. This pocket, containing the hydrogen bond acceptor, is crucially responsible for the COX-2 selectivity [5,52,53]. A similar binding mode could also be observed for other coxibs and non-selective NSAIDs [50]. 

Hydrogen bonding between the sulfonyl group and amino groups Phe504 and Arg499 could, respectively, be observed in 2.46 Å and 2.19 Å as being as strong as that found between the sulfonamide group of celecoxib and the carbonyl side chain of Gln178 in 2.06 Å and Leu338 in 1.97 Å. This agreed with the previous study [51]. We note that CH-pi interactions of this sulfamoylphenyl ring with Val509 (in 3.87 Å) and Ser339 (in 2.26 Å) were also helpful for conformational stabilization (Figure 2b). Other types of noncovalent interactions of celebcoxib occurred at the binding site and are provided in Table S1. The entrance of the COX-2 channel was where the hydrogen bonding occurred between the fluoromethyl group of celecoxib and Arg106 in 2.33 Å and 2.98 Å, as well as between the pyrazole nitrogen of celecoxib and Tyr341 in 3.24 Å. Regarding the per-residue decomposition of binding energy reported in the previous study, it was reported that Arg106, Leu338, Ser339, Arg499, Phe504, and Val509 are the crucial amino acids and commonly contribute to the binding energy of COX-2/celecoxib complex [54,55]. 

### 2.2. Validation of Docking Protocols

To validate the reliability of three scoring functions, we docked the relaxed pose of celecoxib at the binding site of COX-2 and investigated the top-scored pose ranked by AutoDock, ChemPLP and GoldScore functions. Scoring function in AutoDock4 is a semi-empirical free energy force field [56], whereas GoldScore [57] is the empirical fitness function, originally provided with GOLD Suite [58]. The ChemPLP [59] empirical function uses multiple linear potentials and derives hydrogen bonding terms from the total free energy change [60]. Pose prediction by these scoring functions was examined by the fact that the higher the score of GoldScore and ChemPLP functions (or the more negative value in the estimated binding affinity from AutoDock), the better pose is suggested.

Docking results revealed that the pyrazole and sulfomoylphenyl rings of the best-docked poses were nicely overlaid on those rings of the relaxed pose of co-crystallized celecoxib (Figure 2); therefore, hydrogen bonding interactions with the key amino acids were still preserved. The binding affinity of the best-docked pose of celecoxib estimated by AutoDock, was −11.24 kcal/mol (Table 1), agreed well with the previously reported values ranging from −10.01 kcal/mol [61] to −11.00 kcal/mol [62,63]. The fitness score given by ChemPLP and GoldScore for the best-docked poses of celecoxib was, respectively, 95.56 and 79.07. AutoDock, ChemPLP and GoldScore scoring functions can reproduce the native pose of celecoxib with the RMSD value of 0.68, 0.55 and 0.56 Å, respectively (Figure 2c and Figure S2).

### 2.3. Molecular Docking of Oxyresveratrol Analogues

**Figure 3:**
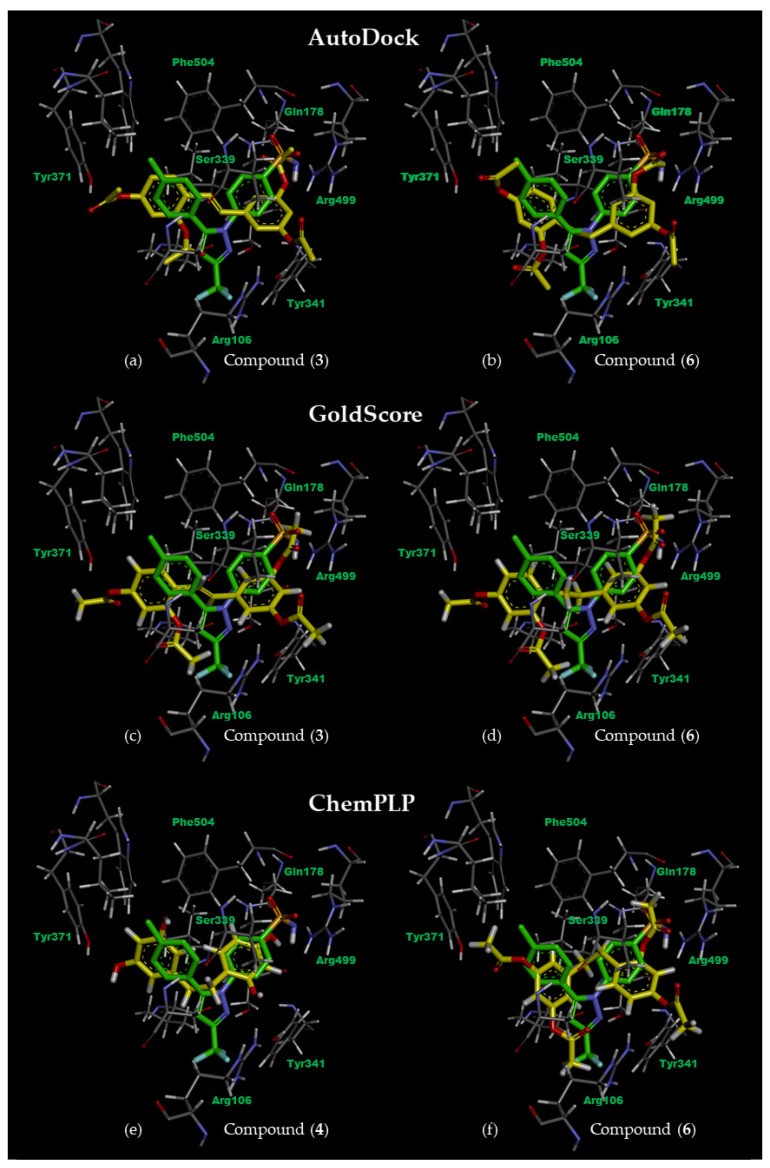
The best-docked poses (yellow) of oxyresveratrol analogues: (**a**) acetoxylated analogue (**3**) of oxyresveratrol and (**b**) acetoxylated analogue (**6**) of dihydrooxyresveratrol ranked by AutoDock; (**c**) compound **3** and (**d**) compound **6** ranked by GoldScore and (**e**) dihydrooxyresveratrol (**4**) and (**f**) compound **6** ranked by ChemPLP—were superimposed onto the relaxed pose of co-crystallized celecoxib (green). Some residues are omitted for clarity.

First, we explore the binding modes of our stilbenoid compounds **1**–**3** and dihydrostilbenoid compounds **4**–**6** by using molecular docking techniques. The best-docked pose of all six compounds was bound in the COX-2 pocket at the same position of the native pose of co-crystallized celecoxib regardless of the applied scoring functions. Hydrogen bonding between Arg499 and the methoxy or acetoxy group of C3 on ring B of oxyresveratrol analogues provides the binding stability, in a similar fashion to that which occurred with sulfonamide of celecoxib (Figure 3). This key interaction was also observed in other coxibs such as etoricoxib and valdecoxib and chromone derivatives bound to COX-2 with high affinity [63]. 

AutoDock ranked the docked poses by the estimated binding energy. In our study, acetoxylated analogues (**3** and **6**) of stilbenoid and dihydrooxyresveratrol were suggested to be the strongest-bound inhibitors for COX-2 with the binding energy of −9.21 and −9.26 kcal/mol compared with −11.24 kcal/mol of celecoxib (Table 1). Figure 3a,b show the binding modes of the best-docked pose of compounds **3** and **6** overlaid with that of celecoxib. Ring B of both compounds was aligned in the disposition of sulfamoylphenyl of celecoxib, while their ring A slipped nearby the position of the pyrazole ring of celecoxib. 

With a similar ranking trend to AutoDock, compound **3** and compound **6** were also the top-ranked ligands suggested by GoldScore fitness function with the docked score of 80.20 and 78.62 regarding that of 79.07 for celecoxib (Table 1). Hydrogen-bonding interactions were observed between Arg499 with acetoxy groups on ring B of the best-docked pose of compounds **3** (Figure 3c) and **6** (Figure 3d). The acetoxy groups on ring B of compound **3** were able to form hydrogen bonds with Arg106 at the binding entrance, however with Tyr341 at the binding entrance in case of compound **6**.

ChemPLP fitness function—the effective docking score of the GOLD docking Suite—predicted the highest score of 83.45 for acetoxylated analogue (**6**) of dihydrooxyresveratrol, against that of 95.56 for celecoxib. Both carboxylate groups on ring A of compound **6** can form hydrogen bonds with Arg499 (Figure 3f) in a similar fashion to sulfonamide of celecoxib. This was no longer observed for dihydrooxyresveratrol (**4**), the second-best ligand suggested by ChemPLP fitness function, with a score of 74.09 (Table 1). Instead, there was a hydrogen bond between the hydroxyl group at C1 of ring A of compound **4** and Ser339 (Figure 3e). ChemPLP also suggested that oxyresveratrol (**1**), was the third-best ligand with the score of 68.34 (Table 1). However, its binding mode was different from the top two ligands, i.e., ring B was interchanged in the side pocket of COX-2 so that one of its hydroxyl groups on this ring formed hydrogen bonds with Gln178 and Leu338.

### 2.4. Biological Activity of Oxyresveratrol Analogues

Oxyresveratrol was successfully extracted and isolated from *Artocarpus lacucha* Buch.-Ham. It was chemically modified to obtain the methoxylated and acetoxylated analogues including those in a series of hydrogenated form with excellent yields.

The in vitro COX-2 inhibitor screening assay and cytotoxicity (CC_50_) in normal MRC-5 cell lines strongly indicates that dihydrooxyresveratrol (**4**) and its parent oxyresveratrol (**1**) are the potent lead among other compounds studied with the IC_50_ values of 11.50 ± 1.54 and 14.50 ± 2.04 (Table 1). Compared with the IC_50_ value of 0.09 ± 0.01 µM for celecoxib, oxyresveratrol (**1**) and its stilbenoid derivatives with methoxy (compound **2**) and acetoxy (compound **3**) substituents showed their IC_50_ values of 14.50 ± 2.04, 25.00 ± 2.34, and 23.00 ± 2.02 µM, respectively. Upon hydrogenation, the IC_50_ values for dihydrostilbenoids **4**, **5**, and **6** were, respectively, 11.50 ± 1.54, 18.10± 2.07 and 19.00 ± 2.01 µM, lower than that of their corresponding parent stilbenoids. Therefore, we note the high potency of dihydrostilbenoids for COX-2 inhibition. The most potent compound in the series is dihydrooxyresveratrol (**4**)**,** a reductive form of oxyresveratrol (**1**), due to its character of being a good hydrogen donor with some degree of hydrogenation.

On the MRC-5 cell lines which are normal human fibroblasts, the cytotoxic effect of all analogues was evaluated and compared with the CC_50_ value of >100 µM for doxorubicin as a positive control. The naturally occurring oxyresveratrol (**1**) and its hydrogenated derivative (compound **4**) demonstrated the significant differences in cytotoxicity with CC_50_ values of 57.48 ± 2.34 and 106.02 ± 3.86 µM. In comparison with these polyhydroxy parent compounds **1** and **4**, the methoxy-containing stilbenoid (**2**) with CC_50_ value of 118.21 ± 2.69 µM, the methoxy-containing dihydrostilbenoid (**5**) with CC_50_ value of 130.32 ± 3.04 µM, and acetoxy-containing dihydrostilbenoid (**6**) with CC_50_ value of 120.51 ± 2.37 µM are high in cytotoxicity, although they are highly lipophilic and suitable for further lead optimization. Considering the cytotoxicity of acetoxy-containing stilbenoid (**3**), its CC_50_ value was 77.66 ± 3.07 µM, which was lower than that of doxorubicin. This is probably due to a slow hydrolysis occurring in the cells to release hydroxylated metabolites. The acetoxylated derivatives do not only present some extent of biological activities, but are also a worthy choice for undergoing the structural modification.

### 2.5. Rescoring Oxyresveratrol Analogues by Quantum Mechanical (QM) Binding Energy 

**Figure 4:**
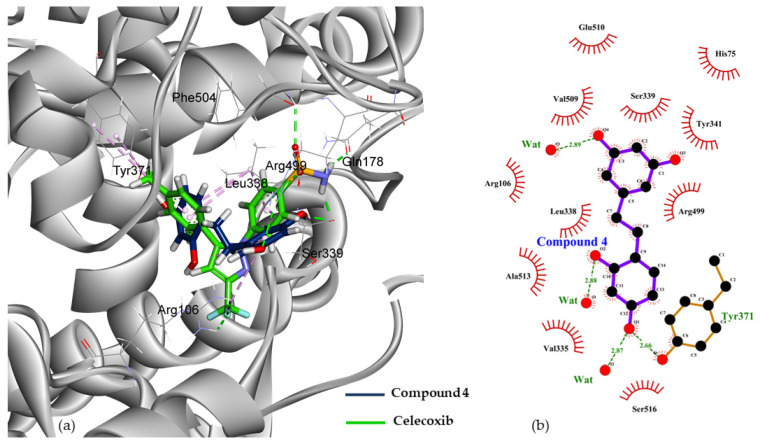
The lowest-QM-energy pose of (**a**) dihydrooxyresveratrol (**4**) (stick representation in navy blue) was overlaid on a native pose of celecoxib (stick representation in green) and in (**b**) its 2D presentation at the active site of COX-2.

Regarding our biological and docking studies, it is difficult to come to a consensus on the structural-activity relationship of oxyresveratrol analogues. However, the binding energy (BE) computed using M06-2X density functional [64] and 6-31G+(d,p) basis set is able to re-score the oxyresveratrol analogues bound to COX-2 considering their biological potency. This computed BE represents the binding affinity of COX-2/ligand complexes, derived from the difference of energy between the bound state of a complex optimized at a M02-2X/6-31G(d) level of theory and the unbound states of both the receptor and ligand according to Equation (2) in the section of computational methods. Among the six non-fluorinated compounds previously discussed, dihydrooxyresveratrol (**4**) was suggested to be the strongest bound ligand in a pocket of COX-2 with the BE of −85.29 kcal/mol in vacuo and of −47.16 kcal/mol in an implicit aqueous solvation (Table 1 and Figure 4). This agreed well with its lowest IC_50_ value of 11.50 µM (Table 1). Nine kcal/mol was compensated by counterpoise (CP) correction for the basis set superposition error on the computed BE of the noncovalently bound complex; therefore, the corrected IEs for the complex of COX-2/compound **4** in vacuo and implicit aqueous solvation were −75.98 and −37.86 kcal/mol, respectively.

**Figure 5:**
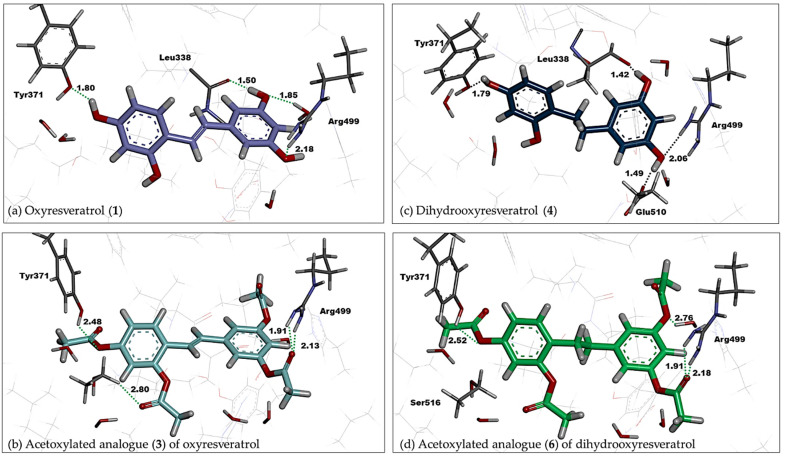
Hydrogen bonding interactions of the lowest-QM-energy poses (stick representation) of (**a**) oxyresveratrol (**1**), (**b**) acetoxylated analogue (**3**) of oxyresveratrol, (**c**) dihydrooxyresveratrol (**4**) and (**d**) acetoxylated analogue (**6**) of dihydrooxyresveratrol in the active site of COX-2.

Interactions of oxyresveratrol analogues with solvated water molecules and key residues of the amino acid at a COX-2 pocket play a role in their binding affinity (Figure 4, Figure 5 and Figure S3). The hydroxyl group at C5 on ring B of compound **4** interacted within 1.42 Å with Ser339, whereas that at C3 of the same ring interacted with Glu510 in 1.49 Å (Figure 5c) and resulted in a slight shift of ring B of compound **4** from the sulfamoylphenyl motif of celecoxib (Figure 4a). It is worth noting that hydrogen bonding interactions within 2 Å between the hydroxyl group positioned at C3 of ring B and Arg499, as well as that at C1 of ring A and Tyr371, occurred not only in the case of compound **4**, but also in oxyresveratrol (**1**) and acetoxylated compounds **3** and **6** (Figure 5). In addition to key interactions with Arg499 and Tyr371, T-shaped pi-pi interactions of aryl ring B of these compounds with His75 and Tyr34 could be observed within 5 Å. For oxyresveratrol (**1**), aromatic pi interactions between its ring A with an alkyl chain of Leu338, Ala513 and Val335 were also observed as well as hydrogen bonding between its hydroxyl groups at C5 of ring B with Leu338 in 1.50 Å (Figure 5a). For acetoxylated analogue (**3**) of oxyresveratrol, the hydrogen bond of its acetoxy group at C3 of ring A with Ser516 could be observed in 2.80 Å (Figure 5b), whereas this occurred with Ser516 in 2.87 Å in the case of the acetoxylated analogue (**6**) of dihydrooxyresveratrol (Figure 5d). 

Hydroxy groups of dihydrooxyresveratrol **4** and its parent compound **1** can serve as either hydrogen-bond donors or hydrogen-bond acceptors, whereas methoxy groups of compounds **2** and **5** can function only as hydrogen-bond acceptors. The more negative the value of the ligand binding to COX-2, the greater the affinity of the receptor-ligand established at the binding site, indicating that the hydroxyl substituent considerably affects the ligand stability upon binding. This can be supported by other computational evidence that reported that the number of phenolic groups of ligand compounds and the ligand size influence their binding activity [65]. Considering the basis set superposition error upon noncovalent binding, we performed CP correction on the optimized complex geometries to obtain the corrected BE. From Table 1, the corrected BE of oxyresveratrol (**1**) was −68.47 kcal/mol in vacuo and −24.84 kcal/mol in implicit aqueous solvation, whereas that of its methoxylated stilbenoid (**2**), was −47.30 and −15.98 kcal/mol, respectively. Strong affinity, however, was observed for acetoxylated compounds **3** and **6,** although their acetoxy groups act as hydrogen-bond acceptors, similar to the methoxy groups. It has been suggested that the acetoxylated analogue (**6**) of dihydrostilbenoid tightly binds to COX-2 with the corrected BE value of −70.34 kcal/mol in vacuo and −24.88 kcal/mol in implicit solvation, which is considerably stronger than that of −55.04 kcal/mol and −25.76 kcal/mol for celecoxib (Table 1). This arose from the fact that the acetoxy group is bulky, highly versatile and able to capture interactions at the binding site. A functional group with bulkiness that has rapidly transformed into a hydrophilic functional group through metabolism in circulation exhibits a good topical anti-inflammatory activity [66]. Therefore, binding affinities of polyphenolic compounds **1** and **4**, as well as acetoxylated compounds **3** and **6**, were found to be much stronger than that of the corresponding methoxylated compounds **2** and **5**. The methoxylated analogues have also been reported in the literature as having poor inhibitory activity against COX-2 [67].

In brief, a series of dihydrogenated compound analogues (compound **4**, **5** and **6**) appears to be superior to the stilbenoid analogues (compound **1**, **2** and **3**) considering both of their computed binding energetics and biological activities (Table 1).

### 2.6. Fluorinated Analogues

**Figure 6:**
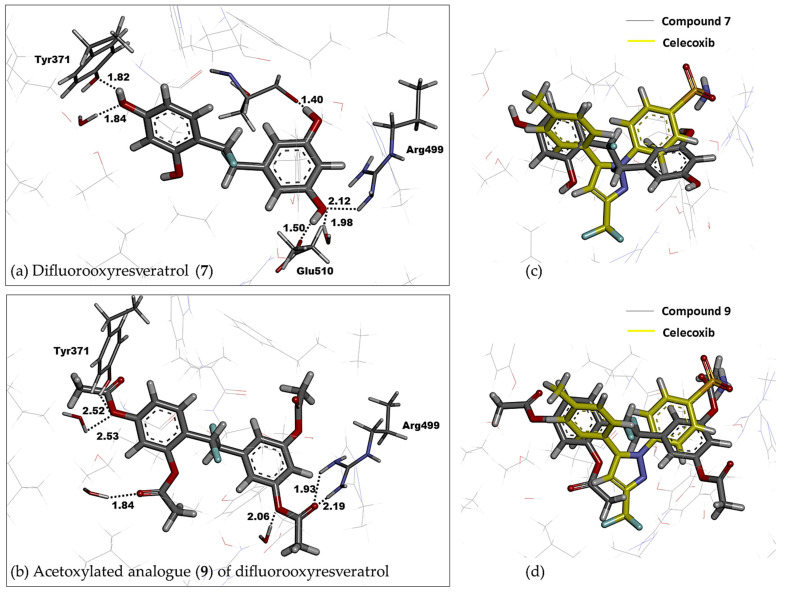
The overlay of the lowest-QM-energy pose of (**a**) difluorooxyresveratrol (**7**) and (**b**) acetoxylated analogue (**9**) of difluorooxyresveratrol (stick representation in grey) on (**c**,**d**) a native pose of celecoxib (stick representation in yellow) in the active site of COX-2.

**Figure 7:**
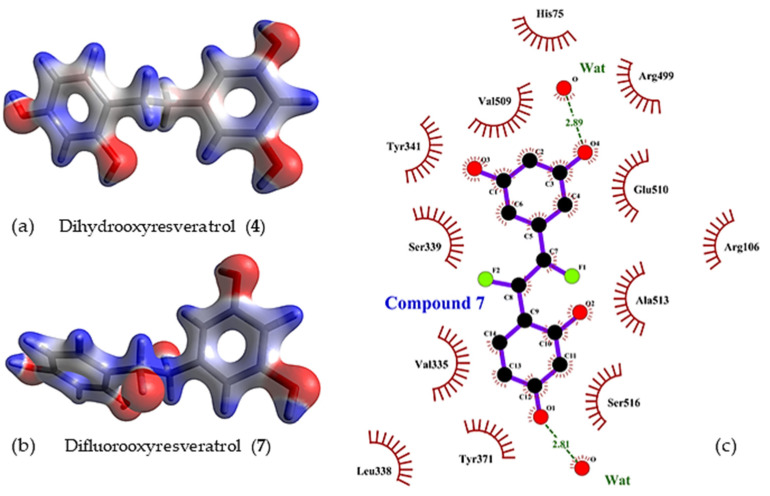
Electron density of (**a**) dihydrooxyresveratrol (**4**) and (**b**) difluorooxyresveratrol (**7**) in (**c**) 2D representation at a COX-2 pocket.

Fluorine-containing compounds have been extensively investigated in drug development. Due to the unique chemical properties of fluorine, fluorine substitution in stilbene-like compounds has been reported to enhance the binding affinity of a compound to a protein target [68,69]. Our computational finding reveals that the incorporation of fluorine with the saturated bridge between both aromatic rings gives a strong affinity towards binding to COX-2. From Table 1, the corrected IEs of difluorooxyresveratrol (**7**) were −78.44 kcal/mol in vacuo and −37.90 kcal/mol in implicit aqueous solvation, the lowest values considering those values of other studied oxyresveratrol derivatives, including fluorinated compounds **8** and **9**. Hydrogen bonding interactions between the hydroxyl group on ring B of compound **7** with Arg499 (Figure 6) within 2 Å was still preserved in a similar fashion to celecoxib, a COX-2 specific NSAID. A negative electron density of difluorine atoms of compound **7** can facilitate its binding affinity without altering any steric bulk (Figure 7).

With a similar binding mode of compound **7**, acetoxylated analogue (**9**) of difluorooxyresveratrol has the corrected BE of −68.65 kcal/mol in vacuo and −24.04 kcal/mol implicit aqueous solvation, which lies in between that of difluorostilbenoid compound **7** and celecoxib (Table 1). This also demonstrates the significance of hydroxyl groups and the fluorine in a chemical structure of our ligands. Fluorine-containing compounds have been reported to have the ability to improve several pharmacokinetic and physicochemical properties and enhance their biological activities [70]. However, our studied fluorinated analogues appear to be cytotoxic to the normal cell lines. We have been investigating the fluorine substitution into oxyresveratrol derivatives to lower their in vitro cytotoxicity with the improved synthetic yield. 

Among our studied compounds, the strongest binding affinity with COX-2 was found to be difluorooxyresveratrol (**7**). Regarding the cell cytotoxicity, we suggest dihydrooxyresveratrol (**4**) is the most potent and the second-best bound ligand for further lead optimization. 

### 2.7. Atomic Charges of Celecoxib and Oxyresveratrol Analogues

The electronic effect of ligand structures upon binding to COX-2 was further examined by considering a distribution of Mulliken atomic charges computed using M06-2X density functional and 6-31G+(d,p) basis set. We found the overall negative charge of carbon atoms on a sulfamoylphenyl ring of celecoxib. This confirmed the fact that the lone-pair electrons of pyrazolic nitrogen of celecoxib can be delocalized to the sulfamoylphenyl motif, which is helpful for its binding stability and anti-inflammatory activity. The positive charge of the sulfur was found to be a value of +1.514e for the unbound state of celecoxib and +0.306e for its bound pose (Table 2), indicating that the sulfonamide group of celecoxib is an electron acceptor, which specifically contributes to a strong interaction with Arg499.

In comparison with celecoxib, a highly positive charge was also observed at C4 on ring B of the unbound pose of oxyresveratrol (**1**), dihydrooxyresveratrol (**4**) and difluorooxyresveratrol (**7**) as shown in Figure S4. It is important to note that only compound **4** preserved a highly positive charge when binding to COX-2, i.e., +0.642e (Table 2 and Figure S5). Our finding suggests that the characteristic of being an electron acceptor upon binding appears to be essential in design selective leads for COX-2 receptor. 

## 3. Statistical Analysis

To investigate the effect of lipophilic substituents: methoxy and acetoxy groups of our oxyresveratrol analogues that have moved towards those with the ability of ligand binding, the absolute values of docking scores obtained from all three scoring functions in Table 1 were averaged out for each compound and compared with that of celecoxib. For any compounds that could remain in the COX-2 pocket as firmly as celecoxib, either their docking scores or estimated binding energetics were expected to be at least comparable to those of celecoxib. Therefore, the insignificance of the mean difference between each compound and celecoxib was statistically analyzed by using the Dunnett t-test, showing the mean square error and standard error of 59.086 and 6.276, respectively (Table S2). At 95% confidence, only acetoxylated analogues, i.e., compound **3**, **6** and **9** showed no significant difference from celecoxib. This highlights the importance of acetoxy groups in ligand structure upon binding. 

To confirm the significance of the mean difference of our computed binding affinity values from that of celecoxib (Table 1), we performed a Dunnett t-test statistical analysis of the average value from all four binding energetics of each compound against that of celecoxib at alpha level of 0.05. With the mean square error of 15.730 and a standard error of 2.804, the BE of the bound compounds **4**, **6** and **7** were found to be significantly different from that of celecoxib at 95% confidence (Table S3). This agreed well with the top rank of dihydrooxyresveratrol (**4**) and difluorostilbenoid (**7**) suggested by the QM re-score.

## 4. Conclusions

Oxyresveratrol analogues can be exploited in the exploration of structurally modified phytochemicals for a novel design of COX-2 inhibitors. We extracted oxyresveratrol from the inner wood of *Artocarpus lacucha* Buch.-Ham. and modified its chemical structure to obtain its methoxylated and acetoxylated analogues, including those in a series of hydrogenated forms with excellent yields and fluorinated analogues. Our compounds exhibit anti-inflammatory potency against human COX-2 with low cytotoxicity to normal cell lines. Docking protocols displayed hydrogen bonds and hydrophobic pi interactions of oxyresveratrol analogues with key residues of COX-2 and some solvated water molecules. With the statistical significance at 95% confidence level of the mean difference, GoldScore and AutoDock scoring suggested acetoxylated analogues as the best leads whereas ChemPLP fitness function ranked dihydrooxyresveratrol (**4**) as the best one, agreed with its potency of IC_50_ against COX-2. Other potential targets that regulate immune responses apart from COX could be further explored and identified by the inverse docking of this compound to members of a protein library and analysis of the similarity of binding patterns that occurred across the protein targets [71].

To predict a more accurate binding affinity, the binding energy of each COX-2/ligand complex was eventually computed at a quantum mechanical level, both in vacuo and with implicit aqueous solvation. The computed results demonstrated that dihydrooxyresveratrol (**4**), the hydrogenated compound, has the strongest binding affinity among non-fluorinated analogues, including its parent oxyresveratrol (**1**). This agreed with the screening results of the biological assay, showing the highest anti-inflammatory activity for dihydrooxyresveratrol (**4**) with very low cytotoxicity to the MRC-5 normal cell line. Analysis of atomic charges highlights the importance of the ligand characteristic of being an electron acceptor on the aromatic ring. Such electrostatic and van der Waals interactions have contributed to the enthalpic component of the binding affinity. However, entropic and solvation effects could also be captured and accounted for ligand-protein binding by free energy calculations such as a free energy perturbation, a linear response approximation (LRA) and a combination of molecular mechanical energies with the Poisson–Boltzmann continuum solvent approaches [72,73]. Incorporation of fluorine into hydrogenated analogues leads to a stronger binding affinity; however, its selective substitution with a low cytotoxicity has been challenging. 

## 5. Experimental Section

### 5.1. Chemistry

Oxyresveratrol (**1**) was chemically modified via semisynthetic pathways to produce stilbenoid compounds **1**–**3** and reductive compounds, dihydrostilbenoid **4**–**6** with high synthetic yields. Firstly, oxyresveratrol (**1**), a naturally occurring compound, was hydrogenated using H_2_ on Pd/C in ethanol at room temperature to afford dihydrooxyresveratrol (2,3′,4,5′-tetrahydroxybibenzyl, **4**). Full *O*-acylations of **1** and **4** were carried out with acetic anhydride in pyridine, achieving compound **3** and **6**, respectively. Full *O*-methyl analog **2** was produced from oxyresveratrol (**1**) with methyl iodide and potassium carbonate in acetone. The hydrogenation of compound **2** was subsequently performed under H_2_ on Pd/C in ethanol at room temperature, giving compound **5**. 

The chemicals and reagents supplied were as follows: palladium on carbon (10% PD, Sigma-Aldrich, St. Louis, MO, USA), Sodium borohydride (fine granulate for synthesis, 96%, Merck, New York, NY, USA), acetic anhydride (ca. 1 mol/L in DCM, TCI, Miami, FL, USA), pyridine (dried with NaOH and distilled before use, Sigma-Aldrich, St. Louis, MO, USA), methyl iodide (ca. 2 M in *tert*-butyl methyl ether, Sigma-Aldrich, St. Louis, MO, USA), K_2_CO_3_ (anhydrous AR, Loba Chemie, Mumbai, India), Hydrogen (ultra-high purity grade, UIG, Skye, Scotland), acetone (Extra dry, 99.8%, AcroSeal, Norwich, UK), ethanol (99% absolute, Fisher Chemical, Waltham, MA, USA). The organic solvents for any extraction were distilled before use. 

The progress of all reactions was monitored on thin-layer chromatography using coated with silica gel 60 (Merck F254). TLC plates were examined under UV light (254 nm and 366 nm) and/or coloring with potassium permanganate stain. Column chromatography was performed on Merck silica gel 60 (0.063–0.200 mm, 70–230 mesh size). The chromatographic solvents were mentioned as *v*/*v* ratios. Air and moisture-sensitive reactions were conducted under an inert atmosphere of argon or nitrogen.

#### 5.1.1. Isolation of Oxyresveratrol from *A. lacucha*

The heartwood of *Artocarpus lacucha* Buch.-Ham. (Synonym *A. lakoocha* Roxb.) was collected after botanical identification. The dried powder of *A. lacucha* heartwood was soaked overnight in ethanol for 72 h prior to each extraction. Three extracts were combined, filtered, and concentrated using a rotary evaporator. The ethanolic crude extract was then purified by column chromatography with 10–15% ethanol in dichloromethane as the eluent to give oxyresveratrol [74].

*Oxyresveratrol* (**1**, *trans*-2,3′,4,5′-tetrahydroxystilbene): pale yellow solid (mp 201.5–202.5 °C); ^1^H NMR (300 MHz, methanol-*d*_4_) δ (ppm) = 7.35–7.31 (m, 1H, Ar*H*), 7.26 (d, *J* = 16.4 Hz, 1H, C=C*H*Ar’), 6.83 (d, *J* = 16.4 Hz, 1H, Ar*H*C=C), 6.47 (d, *J* = 2.1 Hz, 2H, 2 x Ar*H*), 6.34–6.32 (m, 1H, Ar*H*), 6.16 (t, *J* = 2.1 Hz, 1H, Ar*H*) (Figure S6); ^13^C NMR (75 MHz, methanol-*d*_4_), δ (ppm) = 158.1, 157.1, 155.9, 140.8, 127.0, 125.2, 123.5, 116.6, 107.1, 104.3, 102.3, 101.0 (Figure S7); FT-IR (ATR): ν (cm^−1^) = 3500-2600 (br), 1609, 1592, 1518, 1314, 1173, 825, 736. Q-Orbitrap HRMS *m*/*z* [M-H]^+^, calcd for C_14_H_11_O_4_: 243.0657, found: 243.0652.

#### 5.1.2. General Procedure for the Synthesis of Compound **2**

A mixture of oxyresveratrol (**1**, 1.7 g, 7.0 mmol) and potassium carbonate (9.67 g, 70 mmol) in 60 mL of extra dry acetone was added with methyl iodide (35 mL, 70.0 mmol) at room temperature under an atmosphere of argon gas. The mixture was then refluxed for 72 h until the completion of the reaction. The solid in the reaction mixture was filtered off at room temperature. The filtrate was then evaporated under reduced pressure to obtain a yellow oil of crude product. A quick filtration of the crude product was carried out by using a short column with ethyl acetate as eluent. After removal of the solvent and hexane-recrystallization, the desired product of 2,3′,4,5′-tetramethoxystilbene (**2**) was obtained as a white solid [30] with 75% yield, mp 81.2–81.9 °C. ^1^H NMR (300 MHz, CDCl_3_), δ (ppm) = 7.53 (d, *J* = 8.47 Hz, 1H, Ar’*H*), 7.40 (d, *J* = 16.4 Hz, 1H, *H*C=CH), 6.98 (d, *J* = 16.4 Hz, 1H, HC=C*H*), 6.70, (d, *J* = 2.18 Hz, 2H, Ar*H*), 6.54 (dd, *J* = 8.47 Hz, 2.34 Hz, 1H, Ar’*H*), 6.50 (d, *J* = 2.29 Hz, 1H, Ar’*H*), 6.40 (t, *J* = 2.17 Hz, 1H, Ar*H*), 3.90 (s, 3H, OC*H_3_*), 3.86 (s, 9H, 3×OC*H_3_*) (Figure S8); ^13^C NMR (75 MHz, CDCl_3_), δ (ppm) = 161.0 (2×Ar*C*O), 160.7 (Ar*C*O), 158.1 (Ar*C*O), 140.4 (Ar*C*CH), 127.4 (Ar*C*H), 127.0 (*C*H=CH), 123.9 (CH=*C*H), 119.3 (CH*C*Ar), 105.0 (Ar*C*H), 104.4 (2×Ar*C*H), 99.4 (Ar*C*H), 98.5 (Ar*C*H), 55.52 (CO*C*H_3_), 55.56 (3×CO*C*H_3_) (Figure S9); FT-IR (ATR): ν (cm^−1^) = 2999, 2962, 2945, 2912, 2841, 1587, 1570, 1504, 1454, 1421, 1344, 1327, 1313, 1286, 1236, 1193, 1151, 1118, 1105, 1061, 1026, 983, 986, 941, 835, 816, 719, 677. Q-Orbitrap HRMS *m*/*z* [M+H]^+^, calcd for C_18_H_21_O_4_: 301.1440, found: 301.1414.

#### 5.1.3. General Procedure for the Synthesis of Compounds **4** and **5**

Oxyresveratrol (**1**, 1.00 mmol) or compound **2** (1 mmol) was dissolved in 5 mL of ethanol over 10 mol% Pd/C (10.64 mg, 0.10 mmol). The reaction mixture was stirred for 1 h under hydrogen gas in a balloon at room temperature. After the reaction had been complete, the catalyst was filtered off. The filtrate was evaporated under a reduced pressure to provide products **4** or **5** [31].

*2,3′,4,5′-tetrahydroxybibenzyl* (**4**): pale yellow solid (mp 164.5–164.7 °C); quantitative yield; ^1^H NMR (600 MHz, methanol-d_4_), δ (ppm) = 6.81 (d, *J* = 6.5 Hz, 1H, Ar’*H*), 6.31 (s, 1H, Ar’*H*), 6.20 (s, 3H, 3×Ar*H*), 6.12 (s, 1H, Ar’*H*), 2.74 (d, *J* = 4.68 Hz, 2H, C*H_2_*CH_2_), 2.68 (d, *J* = 4.21 Hz, 2H, CH_2_C*H_2_*) (Figure S10); ^13^C NMR (150 MHz, methanol-d_4_), δ (ppm) = 157.7 (2×Ar*C*), 155.9 (Ar*C*), 155.6 (Ar*C*), 145.1 (*C*), 130.1 (Ar*C*), 119.6 (*C*), 106.8 (2×Ar*C*), 106.0 (Ar*C*), 102.1 (Ar*C*), 99.7 (Ar*C*), 36.4 (*C*H_2_), 31.5 (*C*H_2_) (Figure S11); FT-IR (ATR): ν (cm^−1^) = 3361 (br), 2918, 2857, 1603, 1523, 1478, 1398, 1342, 1307, 1283, 1168, 1149, 1106, 979, 966, 855, 833, 809, 689, 602. Q-Orbitrap HRMS *m*/*z* [M+H]^+^, calcd for C14H_15_O_4_: 247.0970, found: 247.0953; [M+Na]^+^, calcd for C_14_H_14_O_4_Na: 269.0790, found: 269.0772.

*2,3′,4,5′-tetramethoxybibenzyl (***5***)*: white solid (mp 44.7–46.8 °C); quantitative yield; ^1^H NMR (600 MHz, CDCl_3_), δ (ppm) = 7.03 (d, *J* = 8.2 Hz, 1H, Ar’*H*), 6.49 (d, *J* = 2.4 Hz, 1H, Ar*H*), 6.43 (dd, *J* = 8.2 Hz, 2.4 Hz, 1H, Ar’*H*), 6.40, (d, *J* = 2.2 Hz, 2H, Ar*H*), 6.34 (t, *J* = 2.3 Hz, 1H, Ar’*H*), 3.84 (s, 3H, OC*H_3_*), 3.82 (s, 3H, OC*H_3_*), 3.80 (s, 6H, 2×OC*H_3_*), 2.88–2.84 (m, 2H, C*H_2_*CH_2_), 2.83–2.79 (m, 2H, CH_2_C*H_2_*) (Figure S12); ^13^C NMR (150 MHz, CDCl_3_), δ (ppm) = 160.7 (2×Ar*C*O), 159.3 (Ar*C*O), 158.4 (Ar*C*O), 145.0 (Ar*C*CH), 130.0 (Ar*C*H), 122.6 (CH*C*Ar), 106.6 (2×Ar*C*H), 103.8 (Ar*C*H), 98.5 (Ar*C*H), 97.8 (Ar*C*H), 55.3 (3×CO*C*H_3_), 36.8 (*C*H_2_), 31.4 (*C*H_2_) (Figure S13); FT-IR (ATR): ν (cm^−1^) = 3005, 2935, 2836, 1588, 1505, 1457, 1430, 1287, 1194, 1150, 1027, 935, 812, 711, 589. Q-Orbitrap HRMS *m/z* [M+H]^+^, calcd for C_18_H_23_O_4_: 303.1596, found: 303.1579; [M+Na]^+^, calcd for C_18_H_22_O_4_Na: 325.1416, found: 325.1397.

#### 5.1.4. General Procedure for the Synthesis of Compounds **3** and **6**

Oxyresveratrol (**1**, 1.00 mmol) or compound **4** (1.00 mmol) was dissolved in 15 mL of anhydrous pyridine. The mixture was stirred at ca. 0 °C in an ice-cold water bath before slowly adding excess acetic anhydride (10 mmol). The reaction mixture was stirred for 2 h. A complete reaction mixture was then quenched with 30 mL of ice-cold water and extracted with 30 mL of ethyl acetate. Three organic extracts were combined before washing with 30 mL of 10% HCl for pyridine removal and 30 mL of water three times. The organic layer was neutralized by 30 mL of sodium bicarbonate, then dried with anhydrous sodium sulfate and filtered afterwards. After the removal of the solvent, the desired product was obtained as compound **3** [30] or **6** [75].

*2,3′,4,5′-tetraacetoxystilbene (**3**)*: white solid (mp 140.8–141.9 °C); 79% yield; ^1^H NMR (300 MHz, CDCl_3_), δ (ppm) = 7.65 (d, *J* = 8.61 Hz, 1H, Ar’*H*), 7.11 (d, *J* = 1.94 Hz, 2H, 2×Ar*H*), 7.05 (dd, *J* = 8.48 Hz, 2.36 Hz, 1H, Ar’*H*), 7.07 (d, *J* = 10.68 Hz, 1H, *H*C=CH), 7.00 (d, *J* = 11.05 Hz, 1H, HC=C*H*), 6.97 (d, *J* = 2.89 Hz, 1H, Ar’*H*), 6.87 (t, *J* = 2.04 Hz, 1H, Ar*H*), 2.38 (s, 3H, C*H_3_*), 2.33 (s, 6H, 2×C*H_3_*), 2.32 (s, 3H, C*H_3_*) (Figure S14); ^13^C-NMR (75 MHz, CDCl_3_), δ (ppm) = 169.0 (2×*C*=O), 168.9 (*C*=O), 168.8 (*C*=O), 151.3 (2×Ar*C*O), 150.5 (Ar*C*O), 148.5 (Ar*C*O), 139.4 (Ar*C*C), 129.5 (Ar*C*C), 127.2 (Ar*C*), 127.1 (Ar*C*), 123.4 (Ar*C*), 119.6 (Ar*C*), 117.1 (2×Ar*C*), 116.4 (Ar*C*), 114.81 (Ar*C*), 21.12 (3×*C*H_3_), 21.01 (O*C*H_3_) (Figure S15); FT-IR (ATR): ν (cm^−1^) = 1753, 1604, 1587, 1577, 1496, 1365, 1292, 1251, 1192, 1147, 1124, 1091, 1014, 975, 889, 846, 833, 815, 775. Q-Orbitrap HRMS *m*/*z* [M+H]^+^, calcd for C_22_H_21_O_8_: 413.1236, found: 413.1210; [M+Na]^+^, calcd for C_22_H_20_O_8_Na: 435.1056, found: 435.1024.

*2,3′,4,5′-tetraacetoxybibenzyl (**6**)*: white solid (mp 72.3–73.3 °C); 78% yield; ^1^H NMR (600 MHz, CDCl_3_), δ (ppm) = 7.19 (d, *J* = 8.33 Hz, 1H, Ar’*H*), 6.95 (dd, *J* = 8.32 Hz, 2.3 Hz, 1H, Ar’*H*), 6.91 (d, *J* = 2.26 Hz, 1H, Ar’*H*), 6.80 (m, 3H, 3×Ar*H*), 2.89–2.85 (m, 2H, C*H_2_*CH_2_), 2.83–2.80 (m, 2H, CH_2_C*H_2_*), 2.33 (s, 3H, C*H_3_*), 2.30 (s, 9H, 3×C*H_3_*) (Figure S16); ^13^C-NMR (150 MHz, CDCl_3_), δ (ppm) = 169.1 (*C*=O), 169.06 (*C*=O), 169.04 (2×*C*=O), 151.0 (2×Ar*C*), 149.30 (*C*), 148.98 (Ar*C*), 143.8 (Ar*C*), 130.4 (2×Ar*C*), 130.3 (*C*), 119.2 (Ar*C*), 119.0 (Ar*C*), 116.1 (Ar*C*), 113.2 (Ar*C*), 35.9 (*C*H_2_CH_2_), 31.3 (CH_2_*C*H_2_), 21.1 (2×*C*H_3_), 21.0 (*C*H_3_), 20.9 (*C*H_3_) (Figure S17); FT-IR (ATR): ν (cm^−1^) = 3488, 2964, 2940, 2368, 1761, 1619, 1590, 1500, 1451, 1371, 1202, 1121, 1135, 1121, 1095, 1014, 976, 897, 845, 828, 782, 703, 686, 661, 594, 526. Q-Orbitrap HRMS *m*/*z* [M+H]^+^, calcd for C_22_H_23_O_8_: 415.1393, found: 415.1378; [M+Na]^+^, calcd for C_22_H_22_O_8_Na: 437.1212, found: 437.1190.

### 5.2. Characterization

Oxyresveratrol and its derivatives were characterized by ^1^H and ^13^C NMR recorded on Bruker NMR-systems (300 and 600 MHz). The deuterated NMR solvents were supplied from Merck for 99.8% chloroform-*d*_1_ and Eurisotop for methanol-*d*_4_. Chemical shift (δ) is given in parts per million ppm referenced to TMS or specified solvent signals. The abbreviations of the signals are used to designate chemical shift multiplicities: s = singlet, d = doublet, t = triplet, q = quartet, m = multiplet, dd = doublet of doublets. Coupling constants are given in Hertz (Hz). ^13^C NMR spectra were measured as ATP- or DEPTQ-experiments. Infrared spectra were collected by using Perkin Elmer infrared spectrophotometer (spectrum GX) and Shimadzu IRAffinity-1S. The melting points were measured on a Kruss Optronic KSP1N apparatus. High resolution mass spectra (HRMS) were recorded on an orbitrap^TM^ Exploris 120 Mass Spectrometer (Thermo Fisher Scientific, Bremen, Germany) using electrospray ionization (ESI) in positive ion mode. 

### 5.3. Measurement of Biological Activities

#### 5.3.1. COX-2 Inhibitory Activity

The in vitro inhibitory of tested compounds were evaluated using a human COX-2 human inhibitor screening assay kit (Item no. 701080) supplied by Cayman Chemicals USA. Celecoxib was used as a positive control. To determine the half-maximal inhibitory concentration (IC_50_), the inhibitory activity of tested compounds was measured in triplicate at each concentration according to the procedure of COX-2 inhibitory immunoassay previously reported [76,77]. COX-2 enzyme and tested compounds at various concentrations were prepared in the reaction buffer (0.1 M Tris-HCl, pH 8.0, containing 5 mM EDTA and 2 mM phenol) containing heme. Prepared solutions were incubated at 37 °C for 10 min and added to arachidonic acid to initiate reactions. COX-derived prostaglandin H_2_ was produced during a 2 min reaction incubated at 37 °C, then quenched with saturated stannous chloride solution. The quenched reactions were incubated at room temperature for 5 min to produce prostaglandin F_2α_. Dilutions were made with the ELISA buffer and measured by using a PGE_2_ ELISA kit according to the manufacturer’s instruction. Absorbance was measured at the wavelength of 405–420 nm by a microplate reader.

#### 5.3.2. Cytotoxicity Assay

The effect of oxyresveratrol and its derivatives on the viability of normal human fibroblasts (MRC-5 cells) were determined by MTT assay. MRC-5 cells were diluted to 2 × 10^5^ cells/mL with a complete medium. The cells (100 µL) were seeded in the wells of the sterile 96-well cell culture plate. After incubation at 37 °C in CO_2_ for 24 h, the cells were treated with different concentrations of sample compounds (100 µL) and doxorubicin as a positive control (100 µL). After incubation for 48 h, MTT solution (20 µL of 5 mg/mL) was added to each well and incubated for 3 h. After that, the complete medium was removed. Then, the cells were lysed with DMSO (100 µL). The yellow MTT dye was reduced by succinic dehydrogenase in the mitochondria of viable cells to purple formazan crystals. After that, absorbance (OD) was measured by a microplate reader at 570 nm. The percentage of cytotoxicity was determined with Equation (1) below [78].

(1) $$Cell\;viability\left(\%\right)=\frac{OD\;of\;treated\;cells}{OD\;of\;control\;cells}\times 100^{\;}$$

## 6. Computational Methods

### 6.1. Preparation of Enzyme Receptor

The x-ray crystallographic structure (PDB entry: 3LN1, chain A) of COX-2 with the resolution of 2.4 Å was retrived from the RCSB protein data bank. Cofactors and hemoglobin were removed from the protein–ligand complex. Missing atoms of amino acids were fixed by using Discovery Studio 4.1 [79]. The nitrogen- and carbon-terminal ends were, respectively, capped with acetoxy and methyl amino groups. The protonation state of amino acids was set at pH of 7.4 by using the PROPKA program [80]. To remove any high energy contacts and relax some of the distorted geometries, restrained molecular dynamics (MD) simulation [81] of chain A of COX-2 with bound celecoxib and 37 crystallized water molecules was carried out for one ns using GROMACS package version 2016.4 [82]. Atomic partial charges of bound celecoxib were detemined by using restrained electrostatic potential (RESP). Its topology for MD simulation was generated using a GAFF force field [83] and AnteChamber PYthon Parser interfacE (ACPYPE) [84], a wrapper script around the ANTECHAMBER package. By using the TIP3P model, the total number of 29,447 water molecules was generated and explicitly included in the system initially neutralized by 0.15 M NaCl. 

### 6.2. Computational Models of Ligand Structures

Geometries of oxyresveratrol and its derivatives were built and optimized by using Gaussian 09 program [85] with M06-2X density functional [64] and 6-31G(d) basis set. For celecoxib, its input coordinate was extracted from the MD trajectories and added to the covalently bonded hydrogen atoms. 

### 6.3. Molecular Docking Protocols 

The automatic docking scheme was applied to the diverse conformations of the oxyresveratrol derivatives in the active site of the COX-2 receptor by using the AutoDock 4.2.5 [56] and GOLD Suite 2020.3 [58]. The size of a grid box was 40 × 40 × 40 Å with the dimensions of 0.375 Å grid spacing. The GOLD docking protocol [86], as previously described, was carried out by using the empirical GoldScore [57] and ChemPLP [59] scoring fitness functions. These two scores of the best-docked pose were examined and compared with the binding energy computed by using AutoDock. The docking performance of each scheme was validated against the crystallographic binding mode of COX-2/celecoxib prior to docking all oxyresveratrol analogues. Protein–ligand interactions were displayed using Discovery Studio 4.1 [79], Chimera 1.11.3 [87] and LigPlot+ v.2.2.4 [88]. The docked ligand-protein complexes predicted by GOLD Suite were then used for quantum mechanical (QM) optimization and calculations.

### 6.4. Computation of QM Energy and Binding Energy (BE)

Five water molecules and twenty amino acid residues: Val74, His75, Arg106, Phe191, Val330, Val335, Leu338, Ser339, Tyr341, Tyr371, Trp373, Arg499, Ala502, Phe504, Val509, Glu510, Ala513, Ser516, Leu517, Leu520 of the COX-2 active site with each of the best-docked ligand poses obtained from the GOLD Suite was quantum-mechanically optimized using M06-2X [64,89] density functional theory (DFT). All dangling bonds were capped with the hydrogen atom. For the complex optimization, we applied the 6-31G(d) basis set to the binding region containing bound ligand and key amino acids: Arg106, Val335, Tyr341, Tyr371, Arg499, and Val509, as well as five water molecules. The lower basis set of 3-21G was applied for the rest. To confirm the binding stability, quantum mechanical (QM) energy of the optimized complexes of COX-2 with bound ligands was computed using M06-2X density functional and 6-31+G(d,p) basis set for both in vacuo and implicit aqueous solvent. The binding energy (BE) was calculated by Equation (2). The basis set superposition error on the calculated BE was also compensated by counterpoise (CP) correction on the optimized complex geometries. We performed all QM calculations using the Gaussian 09 package [85] with the ultrafine integration grid.

(2) $$Binding\;Energy\;(BE)=E_{complex}-\left(E_{bound\_receptor}+E_{bound\_ligand}\right)$$

### 6.5. Statistical Analysis

A one-way analysis of variance was carried out for the docking scores and the estimated binding affinity as a dependent variable. The interaction of a dependent variable and different scoring functions were analyzed using a univariate general linear model with Dunnett’s multiple comparison at the alpha level of 0.05. The dependence of QM energetics on different computational conditions was also investigated in the same fashion. We performed all statistical analysis by using IBM SPSS Statistics version 26 [90]. 

## Figures and Tables

**Table 1:** Docking scores of the best-docked pose, binding energy (BE) computed at the quantum mechanical (QM) level of the optimized COX-2/ligand complexes and the biological activities. The binding affinity and binding energy are in the unit of kcal/mol.

Ligand	AutoDock	GOLD Fitness Function		QM in Vacuo		QM in Implicit Aqueous Solvation		COX-2 Inhibition	MRC-5 Cytotoxicity
	Binding Affinity	GoldScore	ChemPLP	BE (Raw)	BE (CP)	BE (Raw)	BE (CP)	IC_50_/(µM)	CC_50_/(µM)
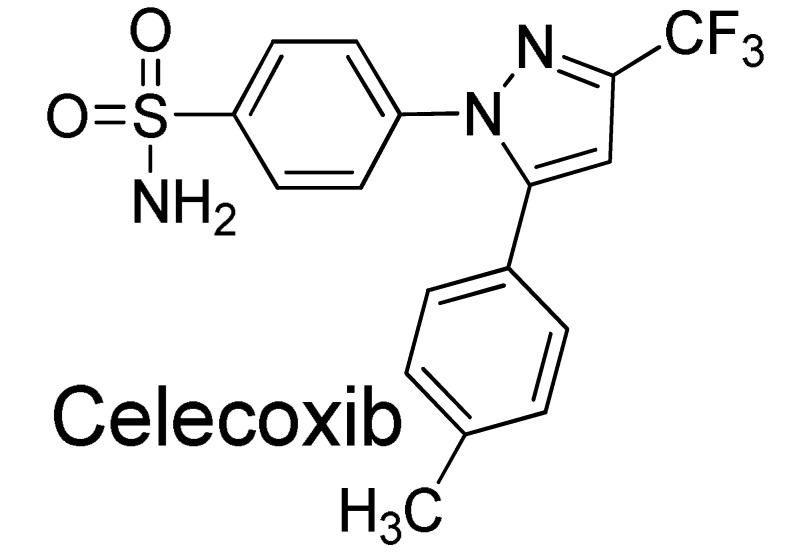	−11.24	79.07	95.56	−65.39	−55.04	−36.11	−25.76	0.09 ± 0.01	n.t. *
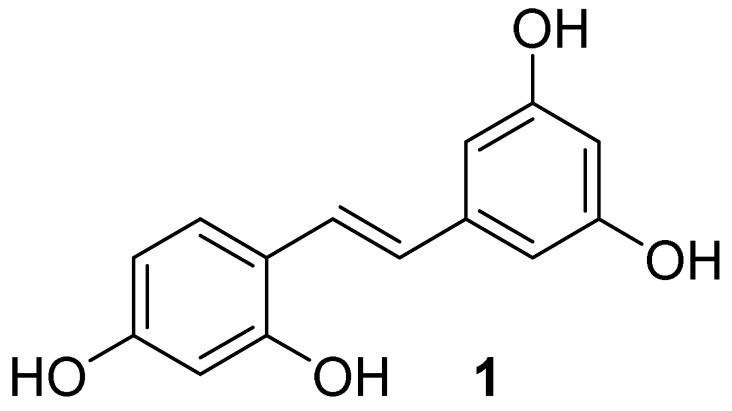	−7.54	55.90	68.34	−77.32	−68.47	−33.68	−24.84	14.50 ± 2.04	57.48 ± 2.34
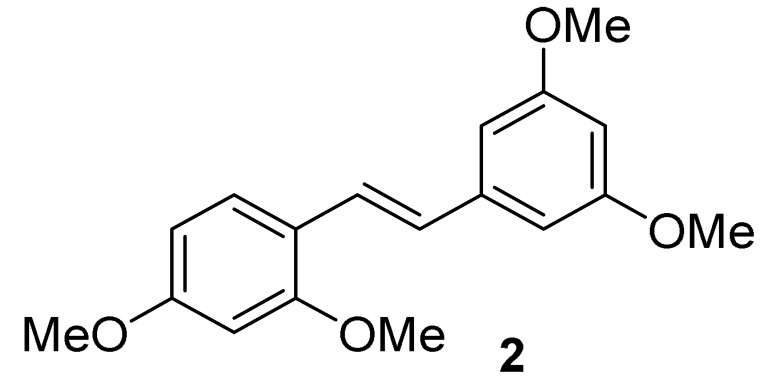	−6.94	59.35	57.63	−56.51	−47.30	−25.18	−15.98	25.00 ± 2.34	118.21 ± 2.69
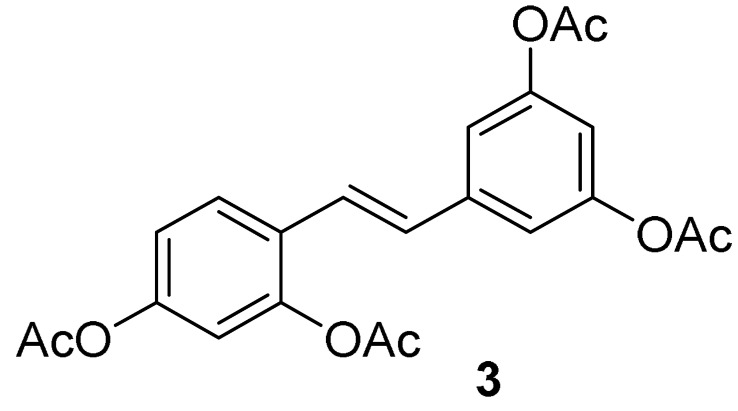	−9.21	80.20	62.08	−80.67	−68.72	−35.36	−23.42	23.00 ± 2.02	77.66 ± 3.07
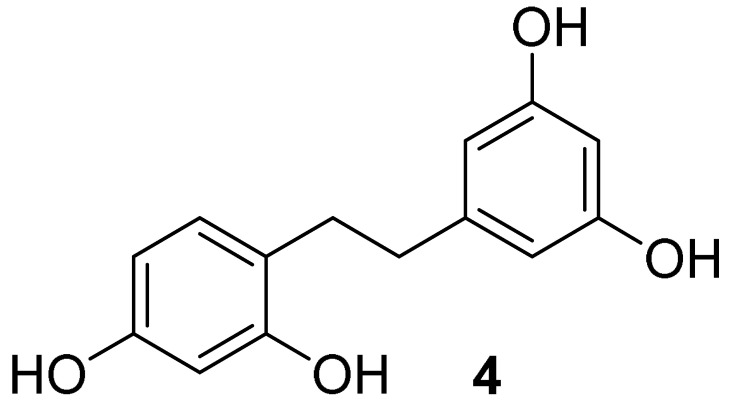	−6.62	56.91	74.09	−85.29	−75.98	−47.16	−37.86	11.50 ± 1.54	106.02 ± 3.86
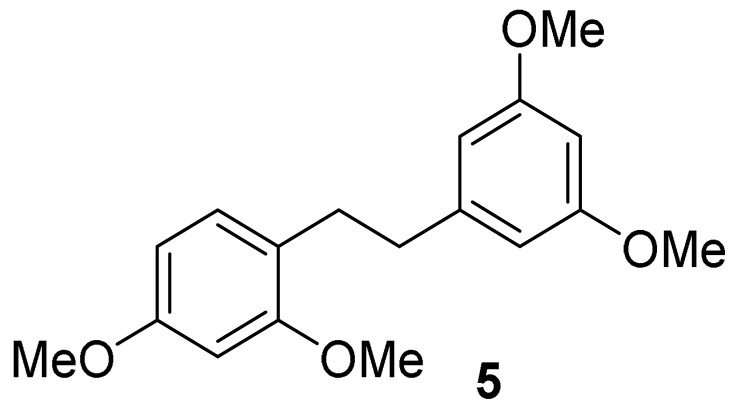	−6.69	60.09	66.57	−58.86	−49.75	−28.78	−19.68	18.10 ± 2.07	130.32 ± 3.04
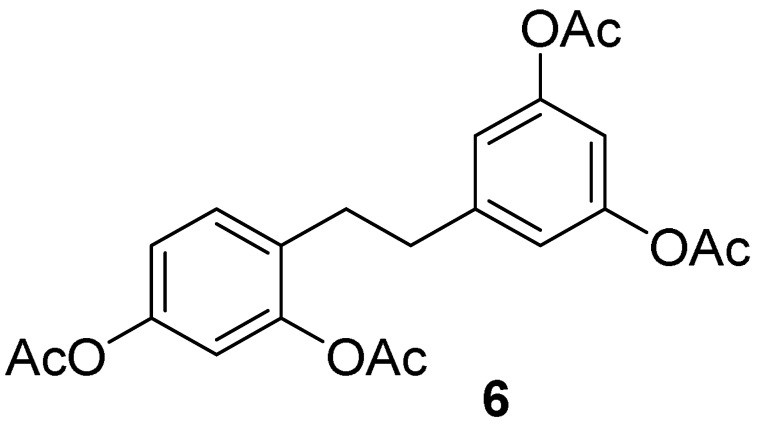	−9.26	78.62	83.45	−82.35	−70.34	−36.89	−24.88	19.00 ± 2.01	120.51 ± 2.37
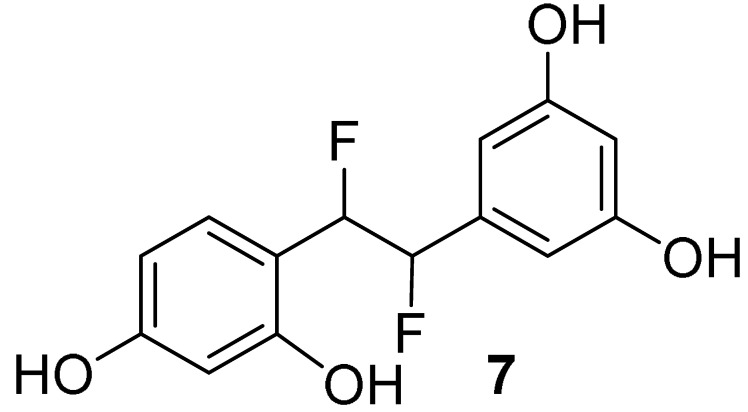	−6.64	58.07	67.66	−87.74	−78.44	−47.21	−37.90	n.t. *	n.t. *
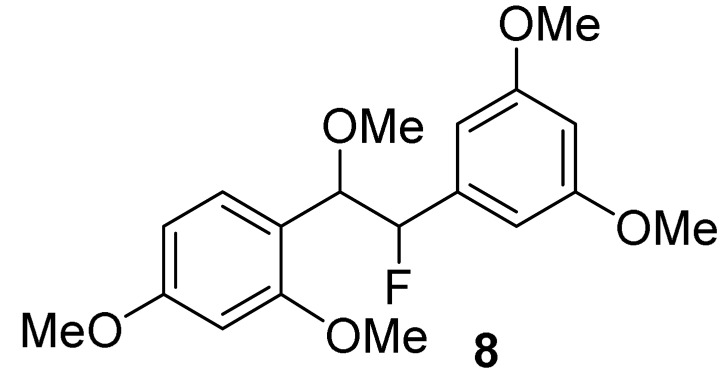	−6.55	63.34	62.82	−55.62	−44.92	−23.28	−12.59	n.t. *	53.04 ± 0.22
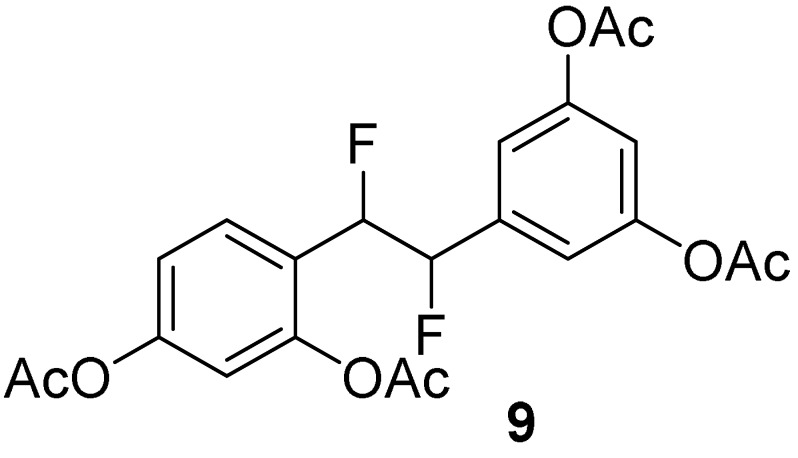	−8.82	78.15	61.22	−81.38	−68.65	−36.78	−24.04	n.t. *	11.69 ± 1.35

* n.t. stands for not tested; IC_50_ and CC_50_ represent the mean ± standard deviation of three replicates.

**Table 2:** Mulliken atomic charges of the aromatic carbon atoms of oxyresveratrol analogues and a sulfur atom, and the electron acceptor in celecoxib.

Ligand	Celecoxib	Ring B	1	2	3	4	5	6	7	8	9
**bound**	S 0.306	C4	−0.539	−0.060	0.111	0.642	−0.308	0.154	0.082	0.021	0.170
		C1	−0.059	0.090	−0.280	0.425	−0.796	−1.064	−0.014	−0.630	−1.538
**unbound**	S 1.514	C4	0.551	0.419	0.363	0.730	0.461	0.476	0.699	0.225	0.108
		C1	0.253	−0.133	0.190	−0.078	−0.509	−0.045	−0.165	−0.204	0.118

## Data Availability

Data analyzed or generated during the study is included in this published article.

## Supplementary Materials

The following supporting information can be downloaded at: https://www.mdpi.com/article/10.3390/molecules27072346/s1, Figure S1: X-ray crystallographic pose of celecoxib bound to COX-2 receptor (PDB entry: 3LN1). Right panel shows the co-crystallized celecoxib and key residues of amino acids at the catalytic site of COX-2; Figure S2: The superposition of the one ns MD structure (grey) and the best-docked poses (yellow) of celecoxib from (a) AutoDock with the RMSD value of 0.68 Å, (b) ChemPLP with the RMSD value of 0.55 Å and (c) GoldScore with the RMSD value of 0.56 Å was within 6 Å of COX-2 binding pocket. Only the amino acid residues in proximity to the binding pocket are highlighted for clarity; Figure S3: Overlay of the lowest-QM-energy poses (stick representation) of celecoxib and oxyresveratrol derivatives in catalytic pocket of COX-2; Figure S4: Distribution of Mulliken atomic charges of unbound pose of (a) celecoxib, (b) oxyresveratrol (**1**), (c) dihydrooxyresveratrol (**4**) and (d) difluorooxyresveratrol (**7**); Figure S5: Distribution of Mulliken atomic charges of bound pose of (a) celecoxib, (b) oxyresveratrol (**1**), (c) dihydrooxyresveratrol (**4**) and (d) difluorooxyresveratrol (**7**) with COX-2; Table S1: Hydrophobic interactions of relaxed pose of celecoxib obtained from MD simulation in the catalytic pocket of COX-2; Table S2: Dunnett t-test of docking scores; Table S3: Dunnett t-test of QM interaction energy; Figure S6: ^1^H NMR (Methanol-*d*_4_, 400 MHz) of oxyresveratrol (**1**); Figure S7: ^13^C NMR (Methanol-*d*_4_, 100 MHz) of oxyresveratrol (**1**); Figure S8: ^1^H NMR (CDCl_3_, 300 MHz) of 2,3’,4,5’-tetramethoxystilbene (**2**); Figure S9: ^13^C NMR (CDCl_3_, 75 MHz) of 2,3’,4,5’-tetramethoxystilbene (**2**); Figure S10: ^1^H NMR (Methanol-*d*_4_, 600 MHz) of 2,3’,4,5’-tetrahydroxybibenzyl (**4**); Figure S11: ^13^C NMR (Methanol-*d*_4_, 150 MHz) of 2,3’,4,5’-tetrahydroxybibenzyl (**4**); Figure S12: ^1^H NMR (CDCl_3_, 600 MHz) of 2,3’,4,5’-tetramethoxybibenzyl (**5**); Figure S13: ^13^C NMR (CDCl_3_, 150 MHz) of 2,3’,4,5’-tetramethoxybibenzyl (**5**); Figure S14: ^1^H NMR (CDCl_3_, 300 MHz) of 2,3’,4,5’-tetraacetoxystilbene (**3**); Figure S15: ^13^C NMR (CDCl_3_, 75 MHz) of 2,3’,4,5’-tetraacetoxystilbene (**3**); Figure S16: ^1^H NMR (CDCl_3_, 600 MHz) of 2,3’,4,5’-tetraacetoxybibenzyl (**6**); Figure S17: ^13^C NMR (CDCl_3_, 150 MHz) of 2,3’,4,5’-tetraacetoxybibenzyl (**6**).

## Sample Availability

Samples of the compounds are available from the authors.
