# Supplementary material for: Computational Analysis and Biological Activities of Oxyresveratrol Analogues, the Putative Cyclooxygenase-2 Inhibitors
Source: Molecules. 2022 Apr 6;27(7):2346. doi: 10.3390/molecules27072346 (PMC9000610; doi:10.3390/molecules27072346)
Supplement: Supplementary file 1 [file molecules-27-02346-s001.zip › molecules-1626055- supplementary.pdf]

Supplementary Information

Computational Analysis and Biological Activities of Oxyresveratrol Analogues, the Putative Cyclooxygenase-2 Inhibitors

Nathjanan Jongkon [1], Boonwiset Seaho [2], Ngampuk Tayana [3], Saisuree Prateeptongkum [2], Nongnaphat Duangdee [3,*] and Panichakorn Jaiyong [2,*]

[1] Department of Social and Applied Science, College of Industrial Technology; King Mongkut's University of Technology North Bangkok, Bangkok 10800, Thailand; nathjanan.j@cit.kmutnb.ac.th

[2] Department of Chemistry, Faculty of Science and Technology, Thammasat University, Pathum Thani 12120, Thailand; boonwiset.seah@dome.tu.ac.th (B.S.); saisuree@tu.ac.th (S.P.)

[3] Drug Discovery and Development Center, Office of Advance Science and Technology, Thammasat University, Pathum Thani 12120, Thailand; ngampuk@tu.ac.th

* Correspondence: duangde@tu.ac.th (N.D.); scpj@tu.ac.th (P.J.)

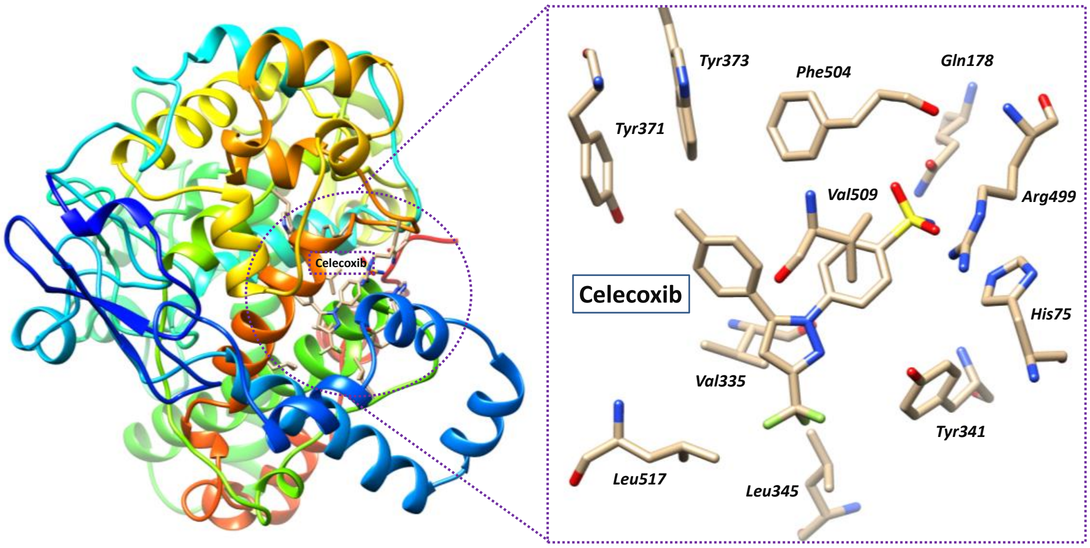

Figure S1. X-ray crystallographic pose of celecoxib bound to COX-2 receptor (PDB entry: 3LN1). Right panel shows the co-crystallized celecoxib and key residues of amino acids at the catalytic site of COX-2.

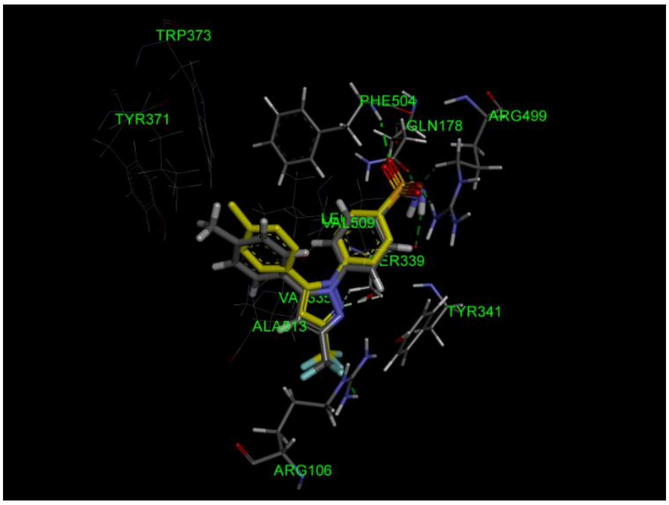

(a)

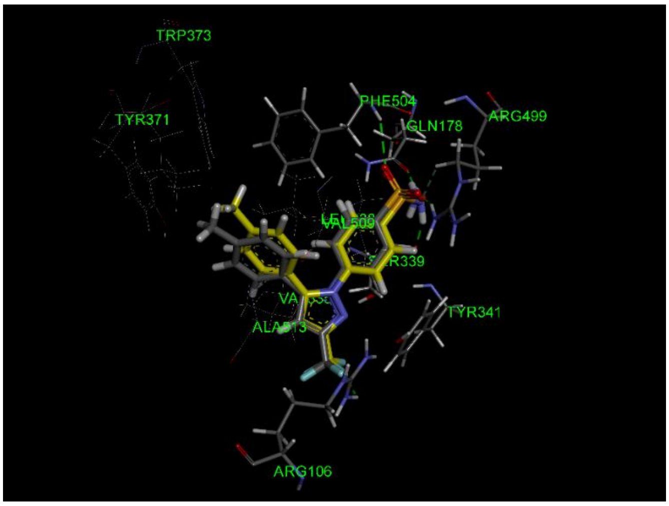

(b)

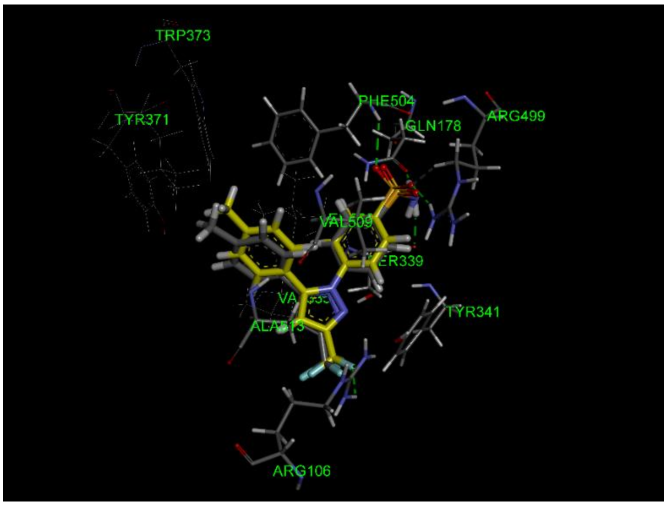

(c)

Figure S2. The superposition of the one ns MD structure (grey) and the best-docked poses (yellow) of celecoxib from (a) AutoDock with the RMSD value of 0. 68 Å, (b) ChemPLP with the RMSD value of 0.55 Å and (c) GoldScore with the RMSD value of 0.56 Å was within 6 Å of COX-2 binding pocket. Only the amino acid residues in proximity to the binding pocket are highlighted for clarity.

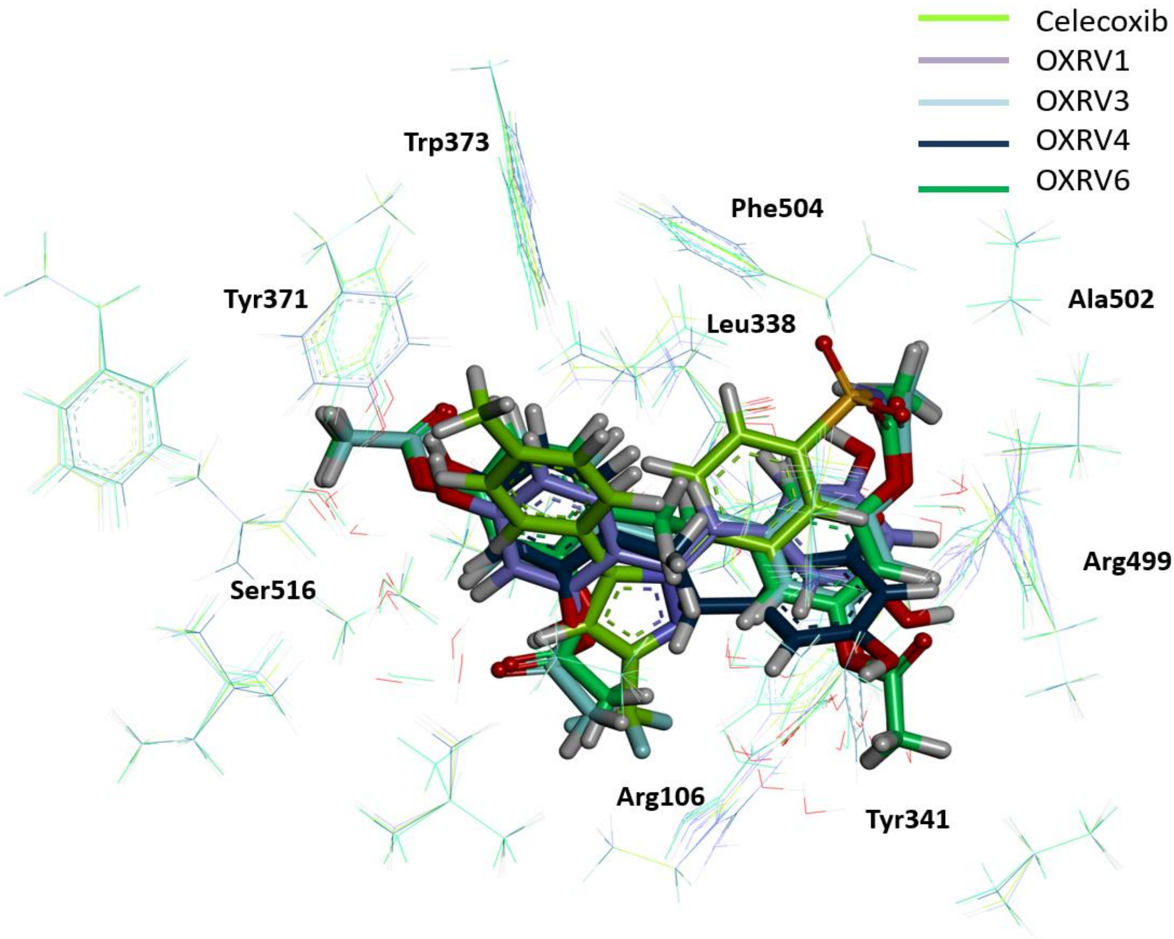

Figure S3. Overlay of the lowest-QM-energy poses (stick representation) of celecoxib and oxyresveratrol derivatives in catalytic pocket of COX-2.

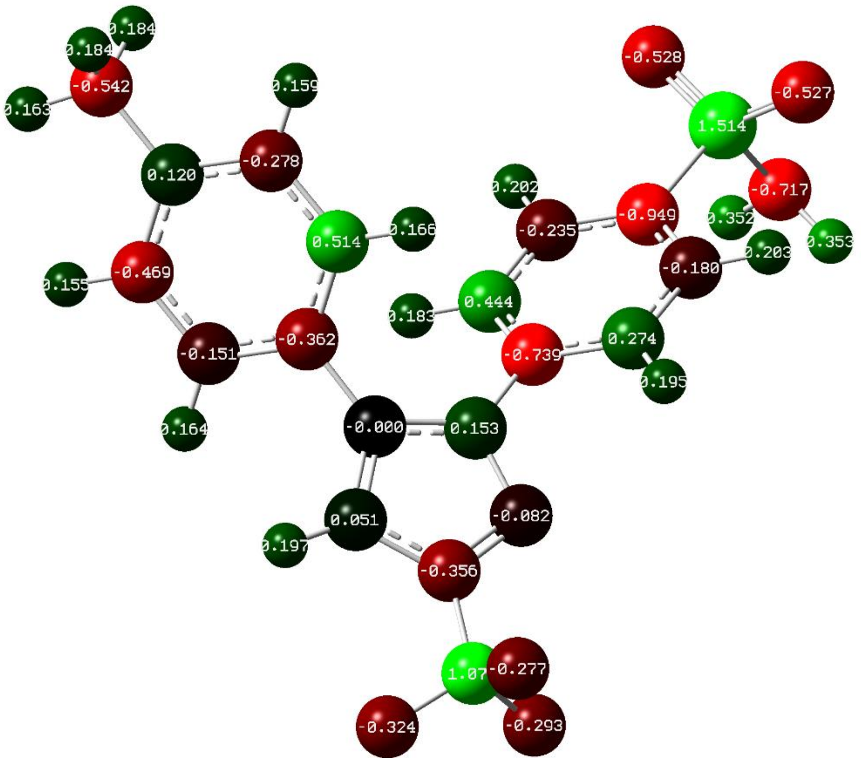
-0.542
-0.528
1.514
-0.278
0.120
-0.949
-0.717
0.514
-0.235
-0.469
-0.180
0.444
0.274
-0.739
-0.362
-0.000
0.153
0.051
-0.356
-0.324
-0.293

(a)

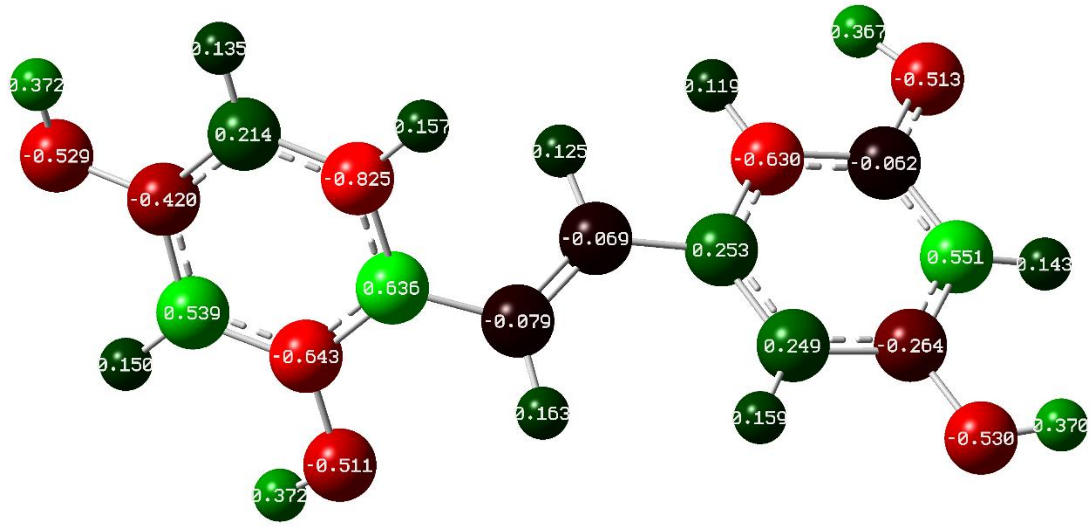
0.135
-0.513
0.214
-0.529
-0.825
-0.630
-0.062
-0.420
-0.069
0.253
0.551
0.539
0.636
-0.079
0.249
-0.264
-0.643
-0.530
-0.511

(b)

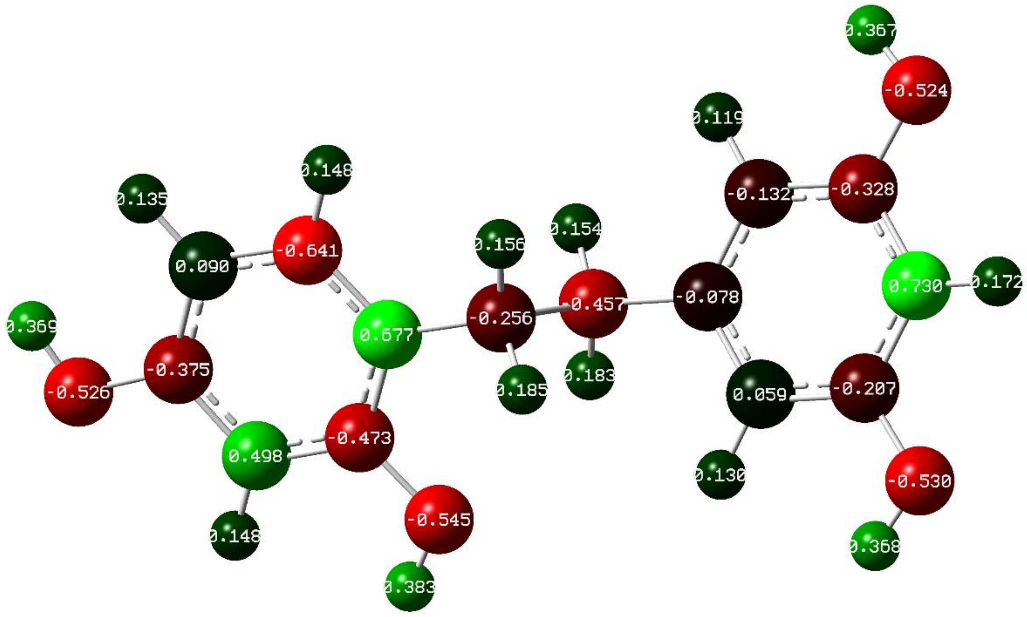

(c)

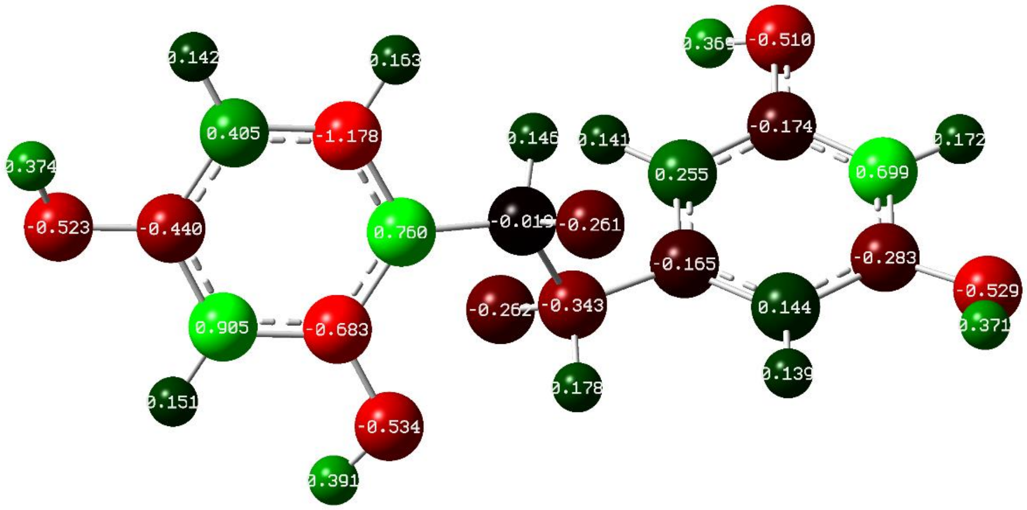

(d)

Figure S4. Distribution of Mulliken atomic charges of unbound pose of (a) celecoxib, (b) oxyresveratrol (**1**), (c) dihydrooxyresveratrol (**4**) and (d) difluorooxyresveratrol (**7**).

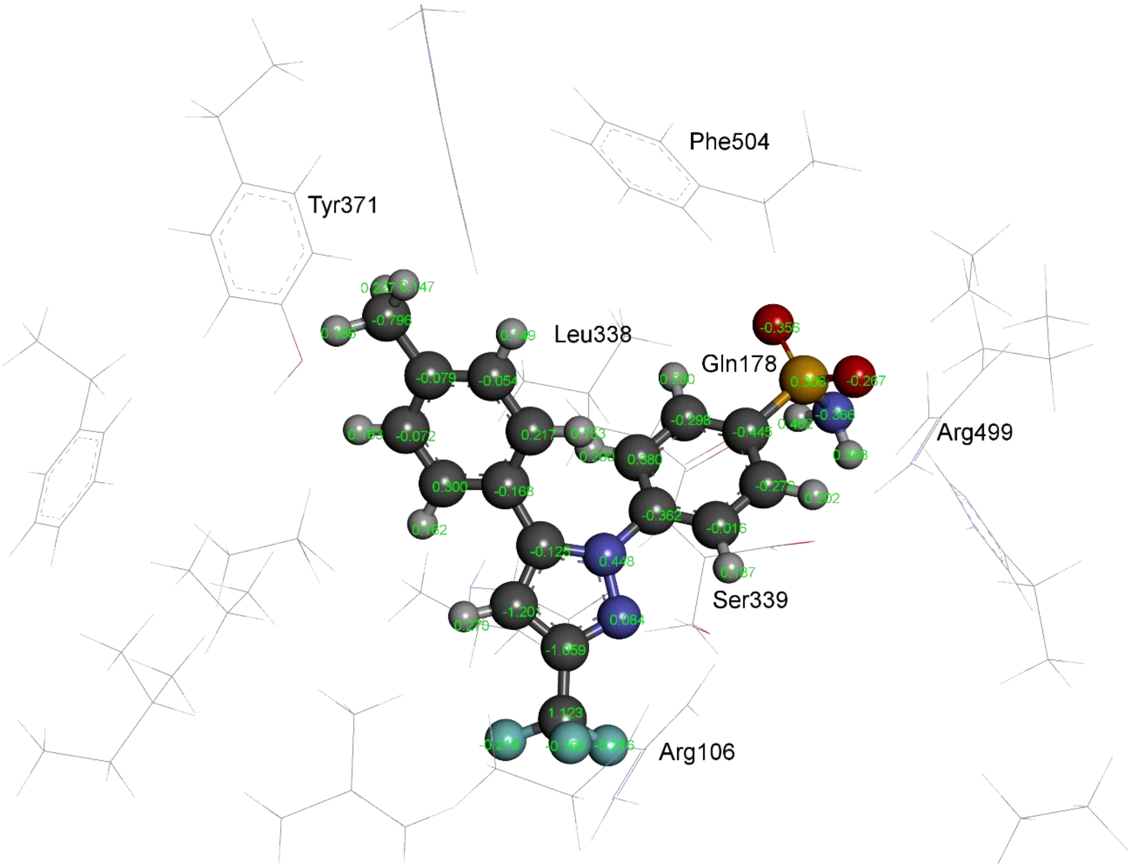
Phe504
Tyr371
Leu338
Gln178
Arg499
Ser339
Arg106

(a)

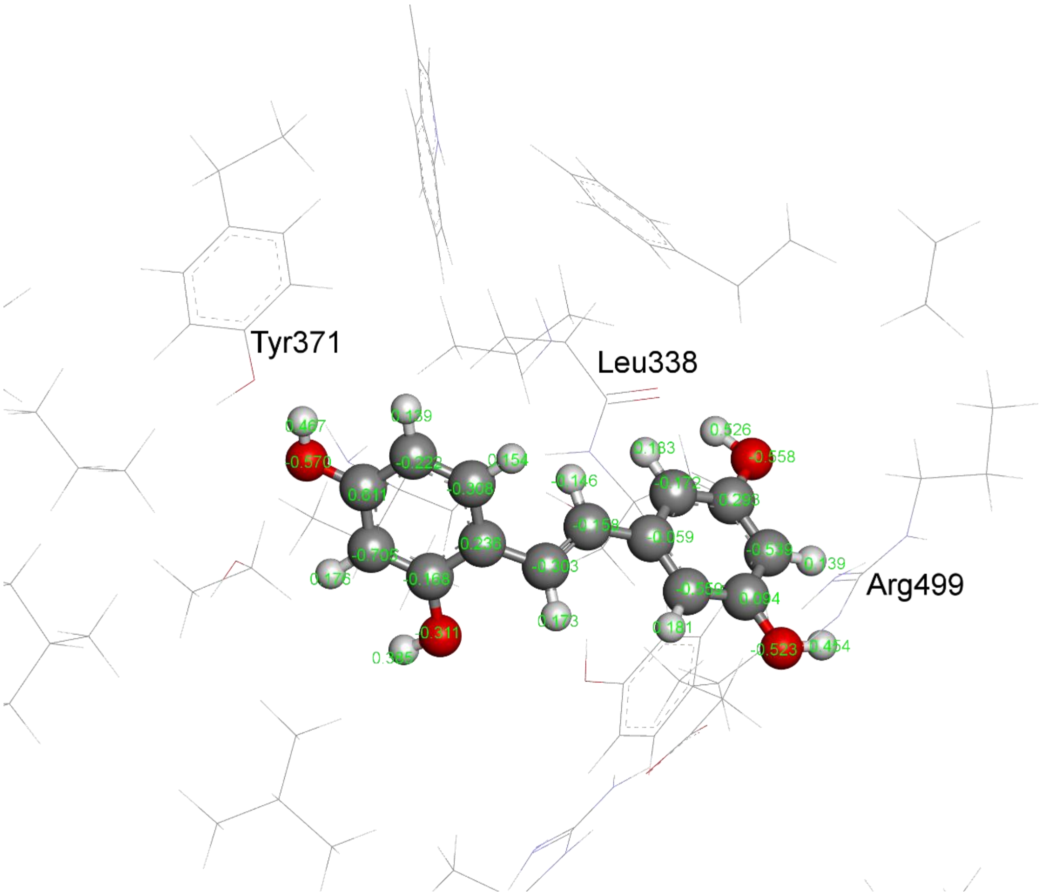
Tyr371
Leu338
Arg499

(b)

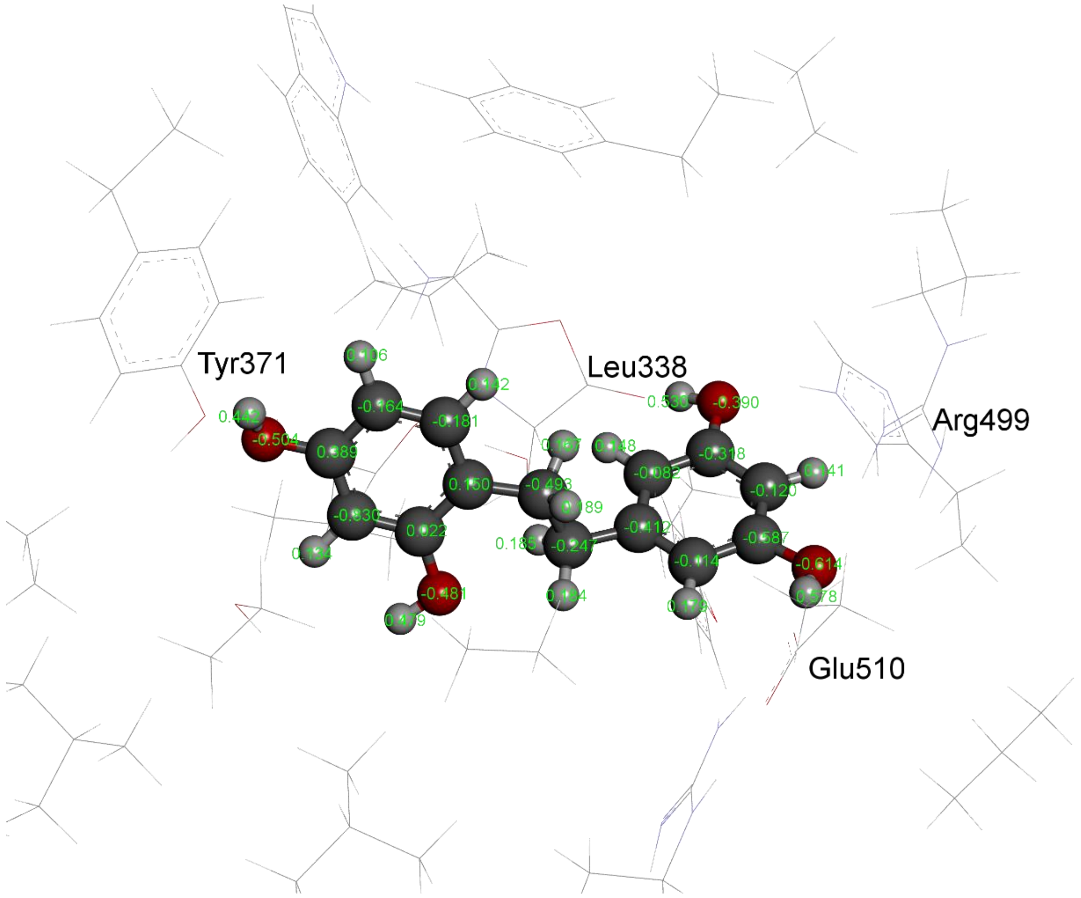

(c)

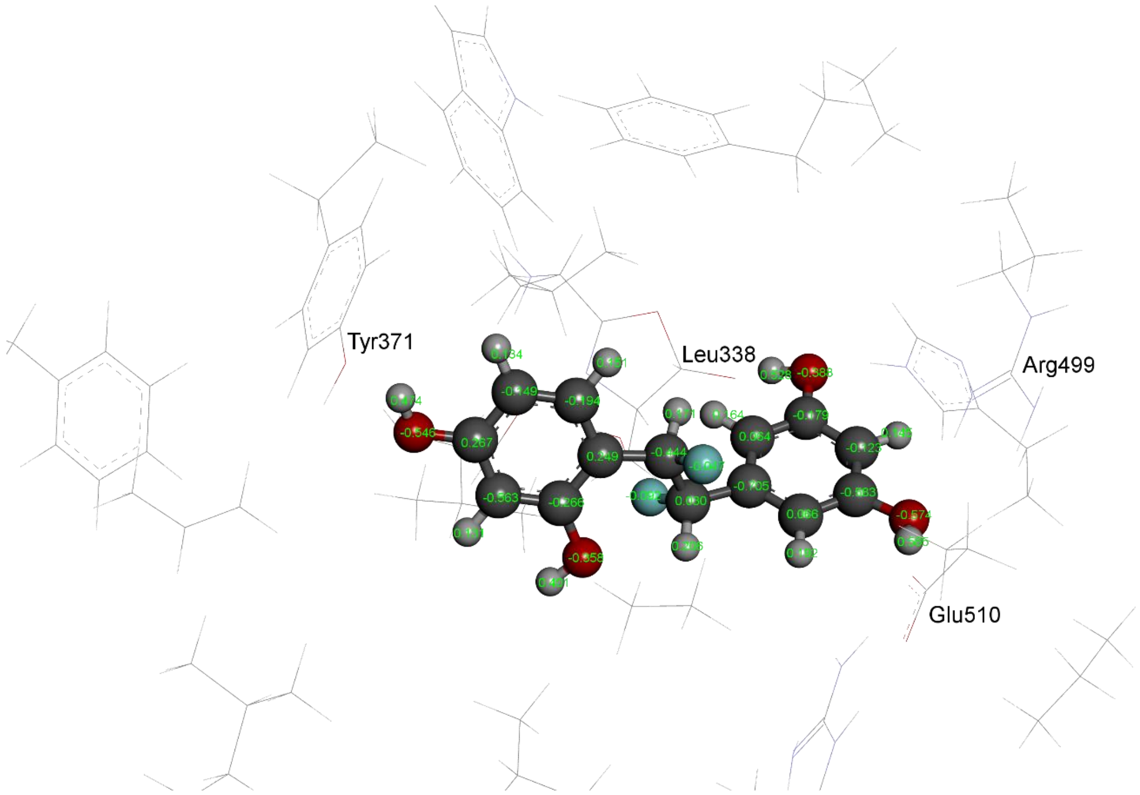

(d)

Figure S5. Distribution of Mulliken atomic charges of bound pose of (a) celecoxib, (b) oxyresveratrol (**1**), (c) dihydrooxyresveratrol (**4**) and (d) difluorooxyresveratrol (**7**) with COX-2.

Table S1. Hydrophobic interactions of relaxed pose of celecoxib obtained from MD simulation in the catalytic pocket of COX-2.

Residues	Distance (Å)	Interaction type
Val335	4.47, 5.34	Pi-Alkyl
Val335	5.27	Alkyl-Alkyl
Leu338	5.38, 5.50	Pi-Alkyl
Ser339	2.26	Pi-Sigma
Tyr341	5.18	Pi-Alkyl
Leu370	5.15	Alkyl-Alkyl
Tyr371	4.57	Pi-Alkyl
Trp373	4.88	Pi-Alkyl
Val509	3.87	Pi-Alkyl
Ala513	3.99, 4.42	Pi-Alkyl

Table S2. Dunnett t-test of docking scores.

Compounds	Mean Difference	Significance	95% Confidence Interval
			Upper Bound
1	-18.03	0.031	-1.571
2	-20.65	0.013	-4.191
3	-11.46	0.195	4.999
4	-16.08	0.056	0.376
5	-17.51	0.036	-1.047
6	-4.85	0.633	11.613
7	-17.83	0.033	-1.374
8	-17.72	0.034	-1.261
9	-12.56	0.149	3.899

Mean square error = 59.086; Standard error = 6.276

Table S3 Dunnett t-test of QM interaction energy.

Compounds	Mean Difference	Significance	95% Confidence Interval
			Upper Bound
**1**	-5.502	0.151	1.652
**2**	9.332	1.000	16.487
**3**	-6.468	0.081	0.687
**4**	-15.998	0.000	-8.843
**5**	6.308	1.000	13.462
**6**	-8.040	0.025	-0.886
**7**	-17.248	0.000	-10.093
**8**	11.472	1.000	18.627
**9**	-7.138	0.051	0.017

Mean square error = 15.730; Standard error = 2.804

Characteristics of oxyresveratrol (1)

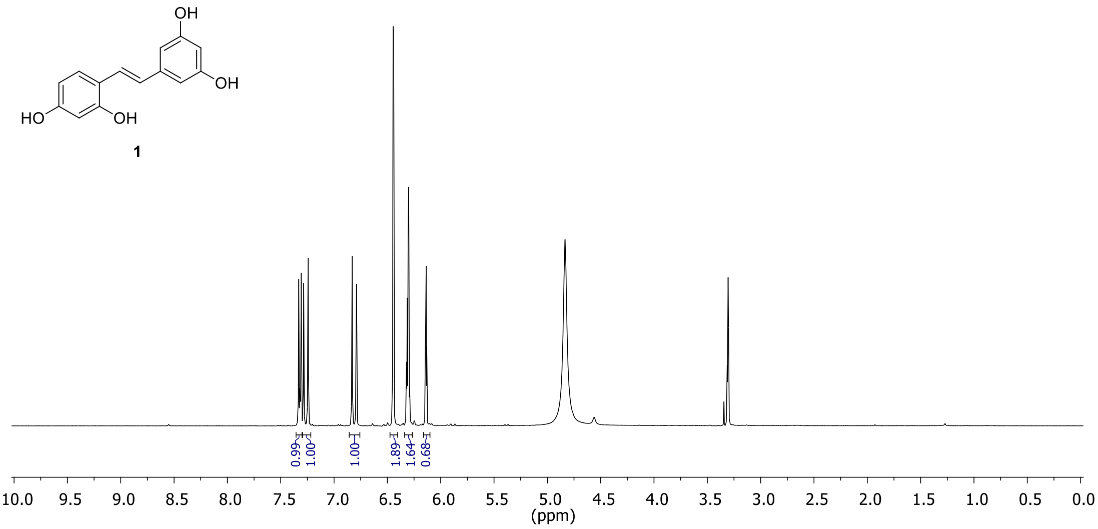

Figure S6 ^{1}H NMR (Methanol-*d*4, 400 MHz) of oxyresveratrol (**1**).

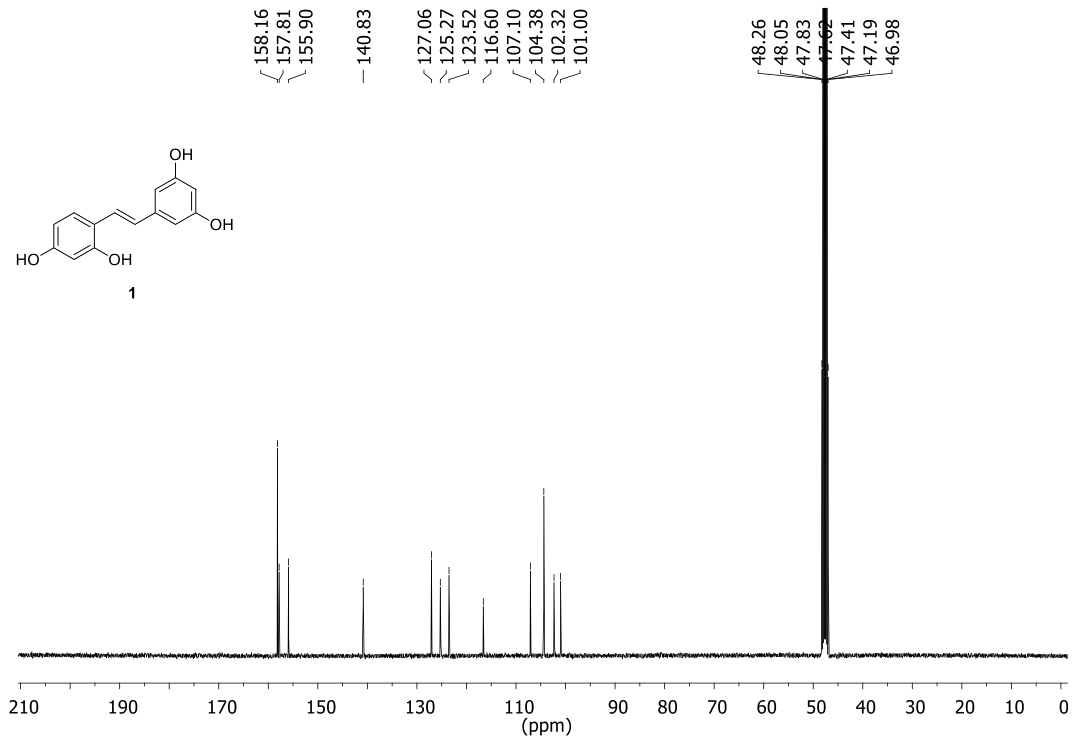

Figure S7 ^{13}C NMR (Methanol-*d*4, 100 MHz) of oxyresveratrol (**1**).

Characteristics of 2,3',4,5'-tetramethoxystilbene (2)

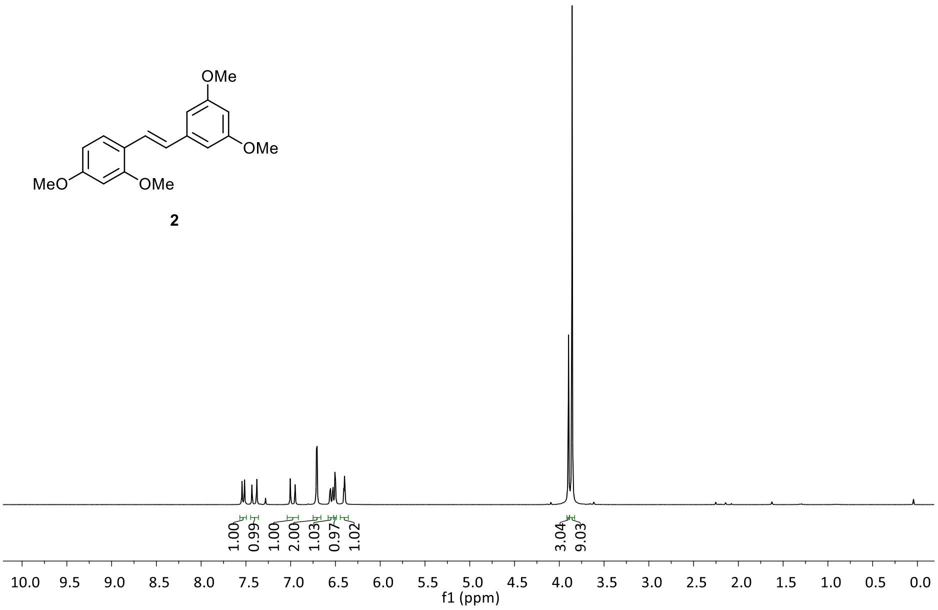

Figure S8 ^{1}H NMR ($CDCl_3$, 300 MHz) of 2,3',4,5'-tetramethoxystilbene (**2**).

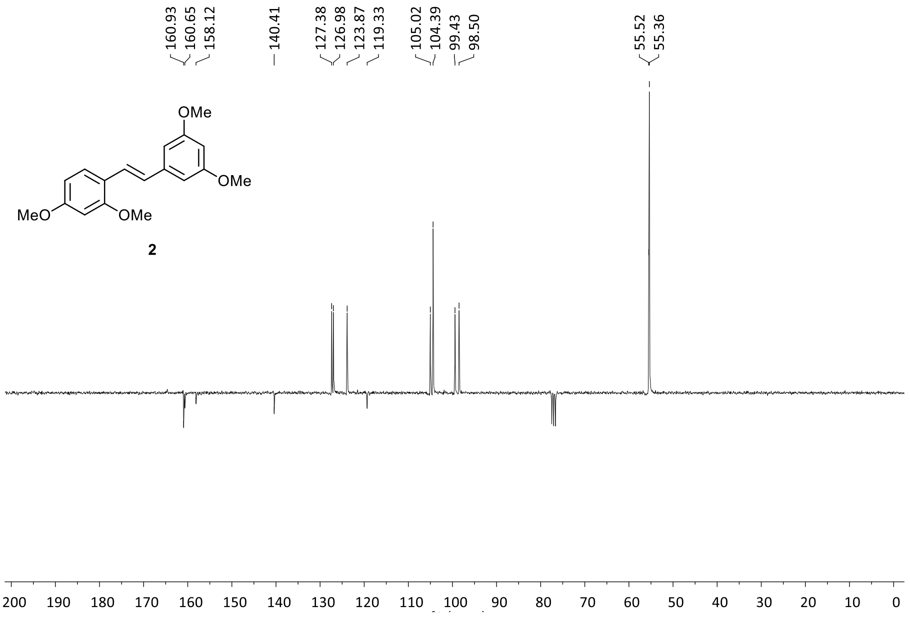

Figure S9 ^{13}C NMR ($CDCl_3$, 75 MHz) of 2,3',4,5'-tetramethoxystilbene (**2**).

Characteristics of 2,3',4,5'-tetrahydroxybibenzyl (4)

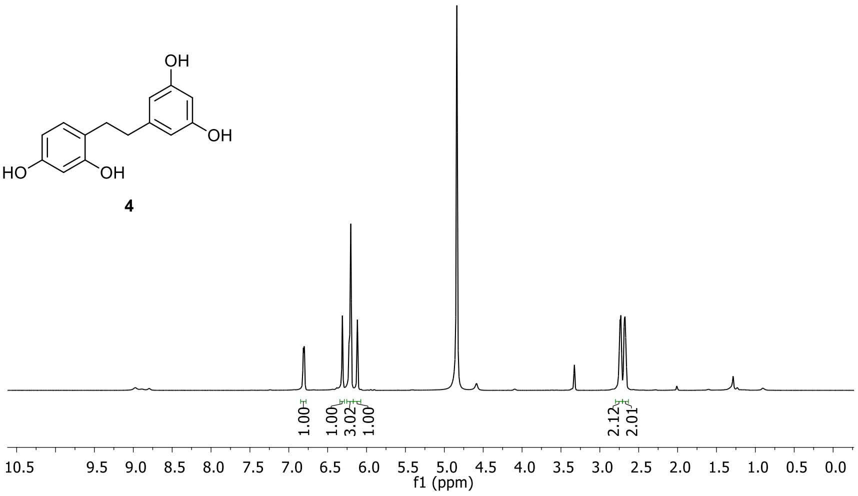

Figure S10 ^{1}H NMR (Methanol-d_4, 600 MHz) of 2,3',4,5'-tetrahydroxybibenzyl (**4**).

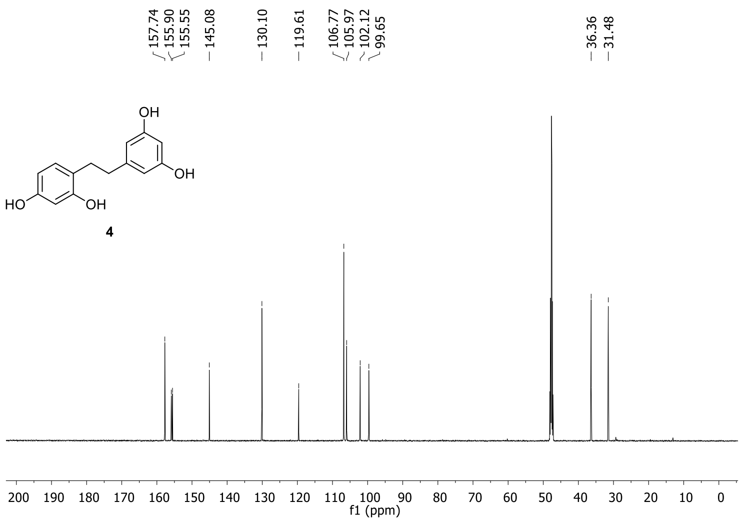

Figure S11 ^{13}C NMR (Methanol-d_4, 150 MHz) of 2,3',4,5'-tetrahydroxybibenzyl (**4**).

Characteristics of 2,3',4,5'-tetramethoxybibenzyl (5)

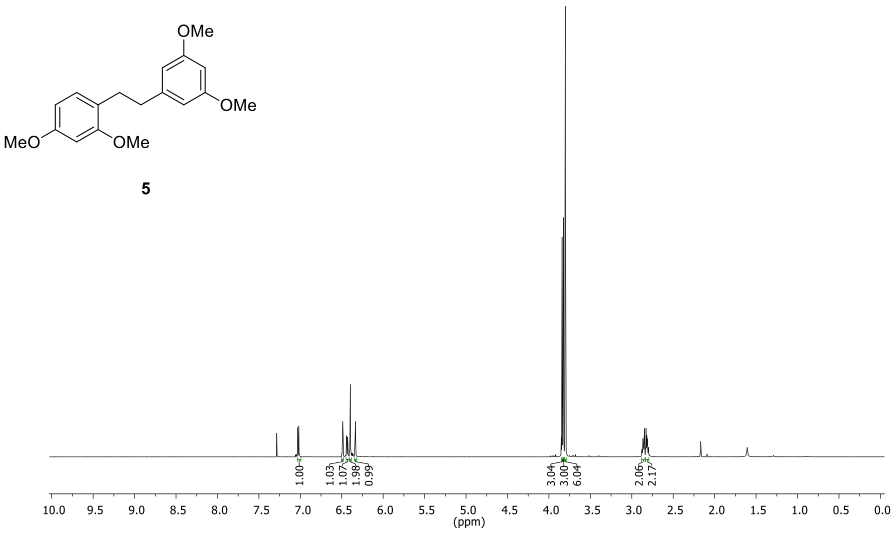

Figure S12 ^{1}H NMR ($CDCl_3$, 600 MHz) of 2,3',4,5'-tetramethoxybibenzyl (**5**).

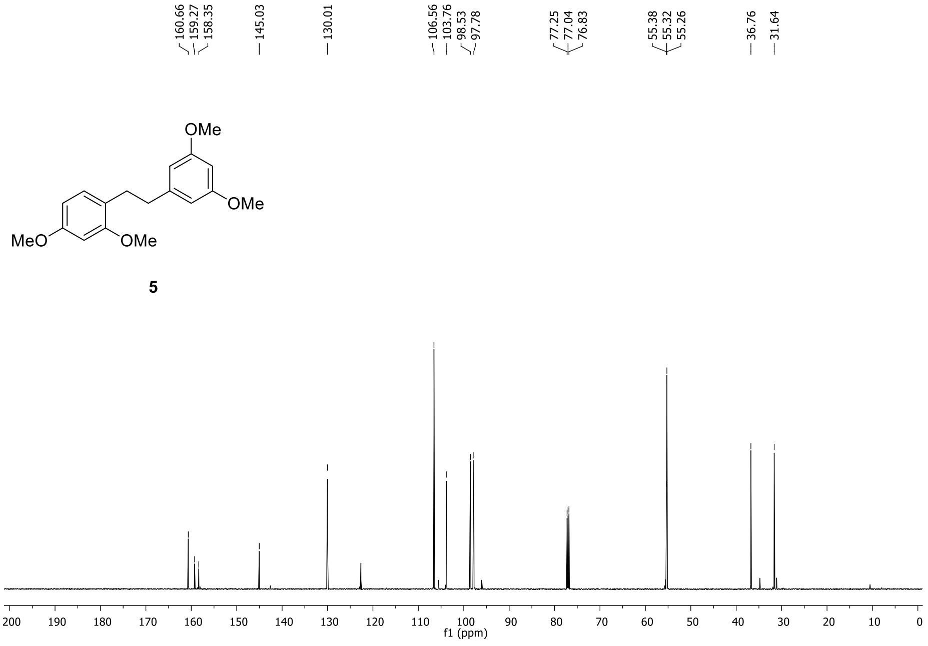

Figure S13 ^{13}C NMR ($CDCl_3$, 150 MHz) of 2,3',4,5'-tetramethoxybibenzyl (**5**).

Characteristics of 2,3',4,5'-tetraacetoxystilbene (3)

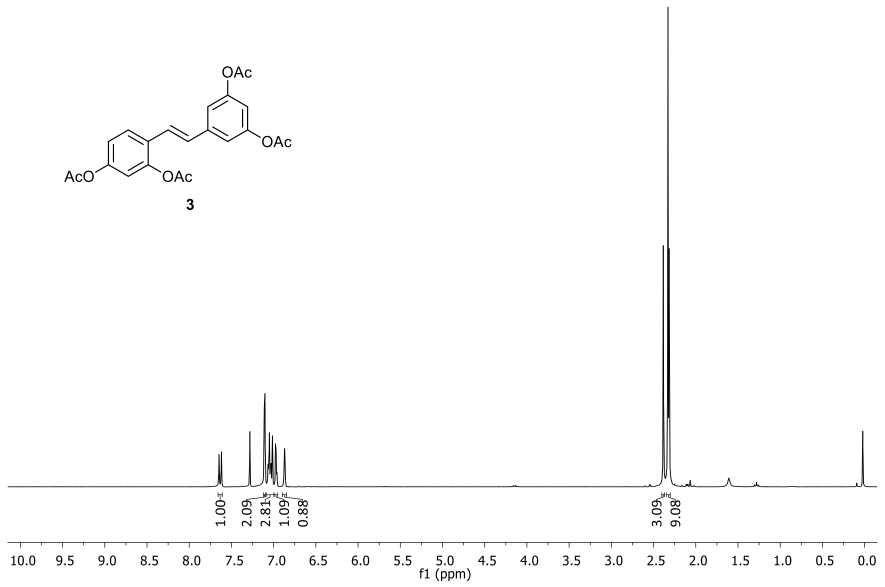

Figure S14 ^{1}H NMR ($CDCl_3$, 300 MHz) of 2,3',4,5'-tetraacetoxystilbene (**3**).

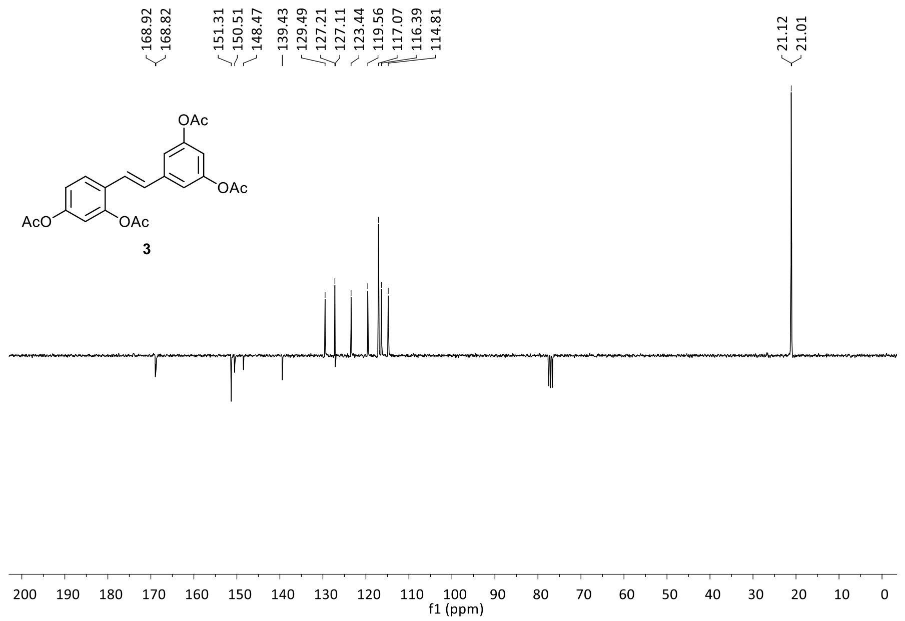

Figure S15 ^{13}C NMR ($CDCl_3$, 75 MHz) of 2,3',4,5'-tetraacetoxystilbene (**3**)

Characteristics of 2,3',4,5'-tetraacetoxybibenzyl (6)

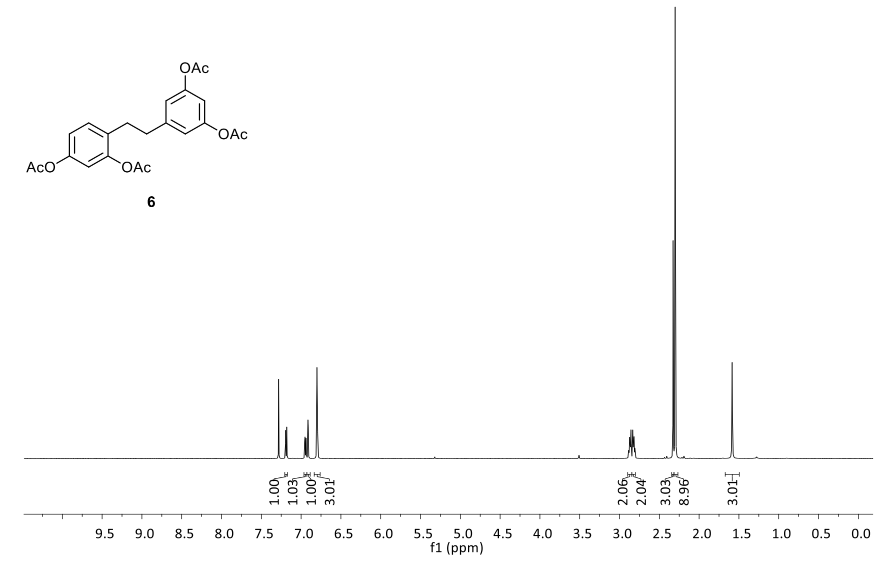

Figure S16 ^{1}H NMR ($CDCl_3$, 600 MHz) of 2,3',4,5'-tetraacetoxybibenzyl (**6**).

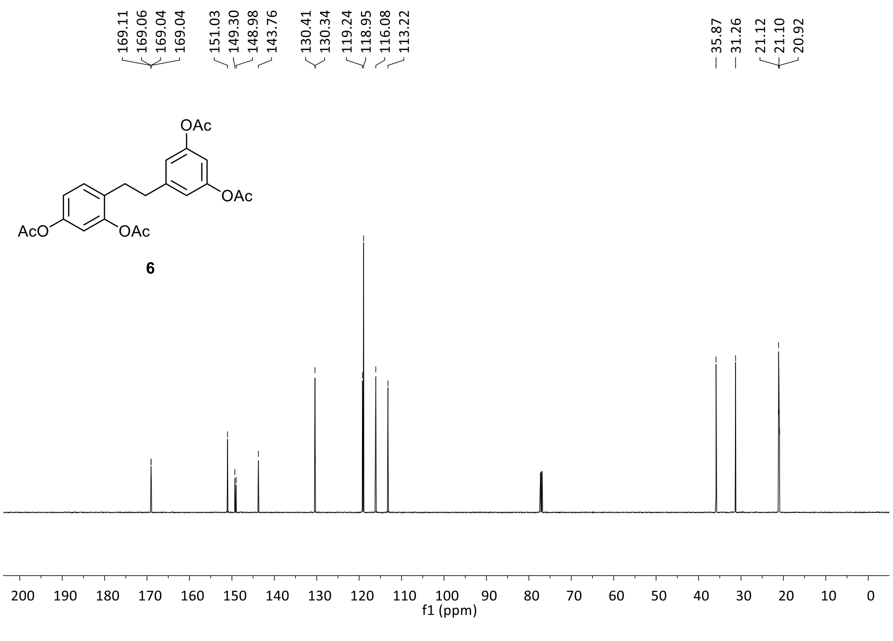

Figure S17 ^{13}C NMR ($CDCl_3$, 150 MHz) of 2,3',4,5'-tetraacetoxybibenzyl (**6**).
